# Anti-Biofouling Polyzwitterion–Poly(amidoxime) Composite Hydrogel for Highly Enhanced Uranium Extraction from Seawater

**DOI:** 10.3390/gels10090603

**Published:** 2024-09-22

**Authors:** Lang Yang, Ye Sun, Yue Sun, Jiawen Wang, Lin Chen, Xueliang Feng, Jinggang Wang, Ning Wang, Dong Zhang, Chunxin Ma

**Affiliations:** 1 State Key Laboratory of Marine Resource Utilization in South China Sea, School of Chemistry and Chemical Engineering, Hainan University, Haikou 570228, China; 21220856000067@hainanu.edu.cn (L.Y.); sunye@hainanu.edu.cn (Y.S.); 21220856000052@hainanu.edu.cn (Y.S.); jiawen10101997@163.com (J.W.); 22110805000014@hainanu.edu.cn (L.C.); 22220856010033@hainanu.edu.cn (X.F.); machunxin@hainanu.edu.cn (C.M.); 2 Key Laboratory of Quality Safe Evaluation and Research of Degradable Material, State Administration for Market Regulation, Hainan Academy of Inspection and Testing, Haikou 570203, China; 3 Key Laboratory of Bio-Based Polymeric Materials Technology and Application of Zhejiang Province, Ningbo Institute of Materials Technology and Engineering, Chinese Academy of Sciences, Ningbo 315201, China; wangjg@nimte.ac.cn; 4 The Wallace H. Coulter Department of Biomedical Engineering, Georgia Institute of Technology and Emory University, Atlanta, GA 30332, USA

**Keywords:** poly(amidoxime), polyzwitterionic hydrogel, anti-biofouling, uranium extraction, seawater

## Abstract

Amidoxime-functionalized hydrogels are one of most promising adsorbents for high-efficiency uranium (U) extraction from seawater, but bioadhesion on their surface seriously decreases their adsorption efficiency and largely shortens their service life. Herein, a semi-interpenetrating zwitterion–poly(amidoxime) (ZW-PAO) hydrogel was explored through introducing a PAO polymer into a poly [3-(dimethyl 4-vinylbenzyl amino) propyl sulfonate] (PDVBAP) polyzwitterionic (PZW) network via ultraviolet (UV) polymerization. Owing to the anti-polyelectrolyte effect of the PZW network, this ZW-PAO hydrogel can provide excellent super-hydrophilicity in seawater for high-efficiency U-adsorption from seawater. Furthermore, the ZW-PAO hydrogel had outstanding anti-biofouling performance for both highly enhanced U-adsorption and a relatively long working life in natural seawater. As a result, during only 25 days in seawater (without filtering bacteria), the U-uptake amount of this ZW-PAO hydrogel can reach 9.38 mg/g and its average rate can reach 0.375 mg/(g∙day), which is excellent among reported adsorbents. This work has explored a promising hydrogel for high-efficiency U-recovery from natural seawater and will inspire new strategy for U-adsorbing materials.

## 1. Introduction

Because of the growing global energy demand, nuclear power has been increasing in the energy mix due to low consumption and environmental protection [1]. However, nowadays the main raw material for nuclear reactors, onshore uranium (U) deposits can only supply the nuclear industry for around 100 years, which poses a severe threat to global energy security [2]. Fortunately for mankind, researchers have found that seawater contains rich U-resources, which are more than 103 times those of proved onshore deposits and can meet human needs for a long time [3]. U-recovery from seawater is therefore essential for the sustainable development of the nuclear energy industry [3]. At present, the strategies of U-extraction include chemical/electrochemical deposition [4], ion exchange [5], membrane separation [6], photocatalysis [7], adsorbing materials [8,9], and so on. However, it is still very difficult to extract U from natural seawater with high efficiency, due to the ultralow U-concentration and many kinds of competitive ions in seawater [10,11].

Among the diverse U-adsorbents including inorganic materials [12,13], organic materials [14], polymers [15], nanoporous-structure materials [16,17], and artificially modified proteins/polypeptides [18], amidoxime-functionalized materials [19] have outstanding overall performance for large-scale U-extraction from seawater in the future. In recent years, various amidoxime (AO)-functionalized adsorbents have been developed (such as fibers [20], membranes [21], porous materials [22,23], and hydrogels [24,25]) for highly efficient U-recovery from seawater, owing to their integration of excellent U-adsorption selectivity and high U-adsorption capacity.

However, nowadays, amidoxime-functionalized materials still cannot achieve large-scale U-extraction from seawater. There main reasons are as follows: besides extremely trace U-concentration and diverse competitive ions in seawater, another important reason is that microorganisms, shellfish, and many other marine organisms in the ocean can adhere onto most kinds of U-adsorbents, resulting in severe biological fouling which reduces U-extraction and even destroys the adsorbent [26,27]. Indeed, although most high-efficiency adsorbents can provide a high U-adsorption capacity from seawater without bacteria and other marine organisms, they cannot maintain a high-efficiency U-adsorption performance from natural seawater without filtering bacteria for a long time [28]. Most recently, to deal with this problem, a few antifouling amidoxime-functionalized U-adsorbents have been explored based on bacteria-killing mechanisms [29,30]. Notwithstanding, these strategies above are just in the early stages of research, and they can only crack microbial cells to kill them and the dead microbes can still adhere onto the surface of adsorbents and hinder the U-extraction process. Therefore, it is still a great challenge to further explore new strategies (such as those based on marine antiadhesion mechanisms [31]) to endow amidoxime-functionalized U-adsorbents with a better antifouling property than before.

**Scheme 1:**
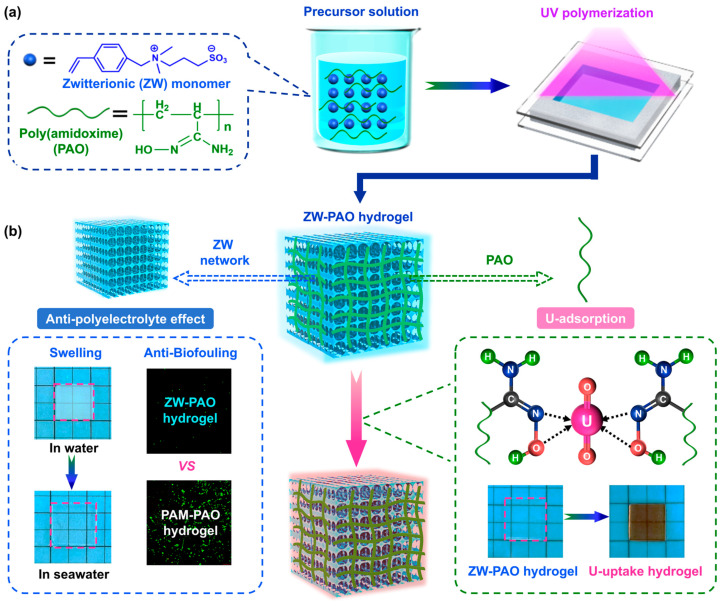
The schematic process of (**a**) the preparation of the ZW-PAO hydrogel and (**b**) selective U-adsorption by the ZW-PAO hydrogel.

In this study, on the basis of our reported semi-interpenetrating polyacrylamide–poly(amidoxime) (PAM-PAO) hydrogels [24] without anti-biofouling properties, a new semi-interpenetrating zwitterion–poly(amidoxime) (ZW-PAO) hydrogel adsorbent with excellent anti-biofouling and highly enhanced U-extraction ability has been developed. The PAO can be synthesized by the amidoxime-functionalization of polyacrylonitrile (PAN). The ZW-PAO hydrogel can be fabricated via UV polymerization through introducing PAO into a precursor solution containing zwitterionic monomer 3-(dimethyl 4-vinylbenzyl amino) propyl sulfonate (DVBAP) at room temperature (Scheme 1a). Firstly, the hydrophilic hydrogel network can effectively disperse and fix the PAO polymer, increasing the contact probability of uranyl ions and amidoxime groups. Furthermore, this semi-interpenetrating ZW-PAO hydrogel has a strong anti-polyelectrolyte effect, which means it can both swell when moved from water to seawater and provide excellent anti-biofouling performance, for highly enhanced U-extraction in real seawater. Most importantly, the outstanding anti-biofouling because of the polyzwitterionic (PZW) hydrogel network can provide both highly enhanced U-adsorption and a relatively long working life in natural seawater (Scheme 1b). This work will provide a hydrogel-based adsorbent with excellent anti-biofouling and U-extraction ability for large-scale U-adsorption from seawater, which will inspire a new strategy to design other kinds of high-efficiency U-adsorbents.

## 2. Results and Discussion

### 2.1. Characterizations of ZW-PAO Hydrogel

**Figure 1:**
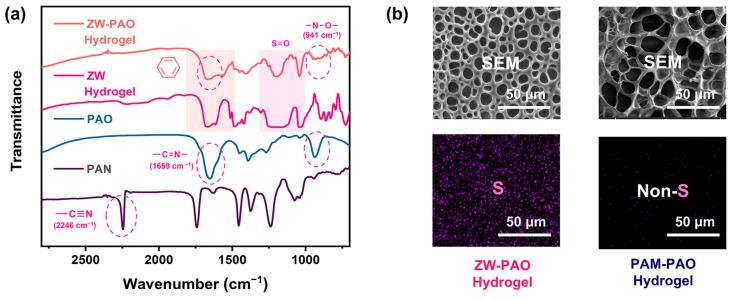
(**a**) The FT-IR spectra of the ZW-PAO hydrogel, PZW hydrogel, PAO, and PAN; (**b**) the EDS mappings of the ZW-PAO hydrogel and PAM-PAO hydrogel.

The synthesized PAO was dissolved under alkaline conditions and dispersed in a monomer solution before polymerization, forming a semi-interpenetrating network in the ZW-PAO hydrogel. The different materials of PAN, PAO, the ZW hydrogel, and the ZW-PAO hydrogel were tested by FT-IR. PAN had a specific peak of the nitrile group (-C≡N) at 2246 cm^−1^ (Figure 1a), which disappeared after the oximation and was replaced by two specific adsorption peaks at 1659 cm^−1^ (-C=N) and 941 cm^−1^ (-N-O). All these changes of specific peaks from PAN to PAO manifested that the PAO had been successfully prepared, indicating the conversion from PAN to PAO. In addition, the characteristic peaks of the benzene ring (1600–1700 cm^−1^), -S=O (1100–1200 cm^−1^), and PAO indicated the successful preparation of the ZW-PAO hydrogel. Polyacrylamide–poly(amidoxime) (PAM-PAO) hydrogels without anti-biofouling properties were prepared as anti-biofouling ZW-PAO hydrogel controls. The morphologies of both the ZW-PAO hydrogel and PAM-PAO hydrogel were further characterized by SEM and EDS mapping images. As shown in Figure 1b, both hydrogels have uniform pore structures, and the distribution of the S element in the ZW-PAO hydrogel proved the successful preparation of zwitterionic polymers. In addition, the average diameter of the ZW-PAO hydrogel was much smaller and denser than that of the blank PAM-PAO hydrogel, which can be explained as follows: the dry mass percentage of the ZW-PAO hydrogel (41.92 wt% in Table S1) was much higher than that of the blank PAM-PAO hydrogel (7.10 wt%) [24].

### 2.2. Swelling Performance of ZW-PAO Hydrogel

**Figure 2:**
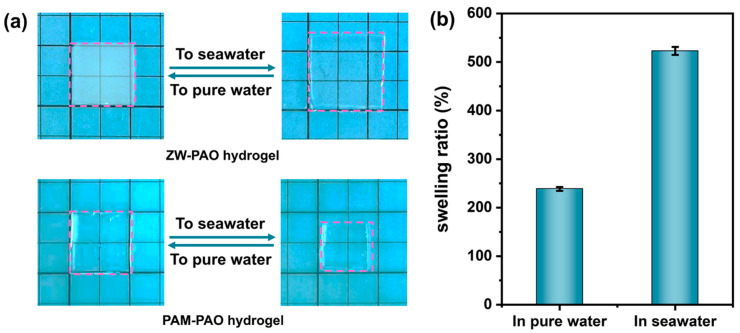
(**a**) The photos of the switching of the ZW-PAO hydrogel and PAM-PAO hydrogel in seawater and pure water; (**b**) swelling ratio of the ZW-PAO hydrogel in pure water and seawater water.

Unlike traditional polyelectrolyte hydrogels, this ZW-PAO hydrogel, as one of the zwitterionic hydrogels, had a strong anti-polyelectrolyte effect [32,33], which means it can swell in seawater and shrink in pure water (Figure 2a). Because of the strong anti-polyelectrolyte effect by paired positively charged -N^+^(CH_3_)_2_- and negatively charged -SO_3_^−^ on the polymeric chain of DVBAP, this polyzwitterionic hydrogel network can become more hydrophilic when moved from pure water to seawater with high ionic strength. Therefore, compared with the ZW-PAO hydrogel in pure water, it can swell to a big size in seawater. In addition, when moved from pure water to seawater, the ZW-PAO hydrogel changed from being translucent white to transparent, because the hydrogel polyzwitterionic network became more and more hydrophilic and the network–water microphase separation gradually disappeared [33]. As a contrast, due to the influence of salt ions, the neutral PAM hydrogels could shrink in seawater. The swelling behavior of the ZW-PAO hydrogel in seawater indicated that it could have better hydrophilicity when moved from pure water to seawater, which was verified by comparison of the air/seawater contact angles between the ZW-PAO hydrogel and the PAM-PAO hydrogel (Figure S1). Compared with the ZW-PAO hydrogel in pure water, the swelling ratio of the ZW-PAO hydrogel in seawater was significantly higher, which further manifested that the hydrogel could be more conducive to U-adsorption in seawater. (Figure 2b, Table S1).

### 2.3. Qualitative U-Adsorption Performance of the ZW-PAO Hydrogel

**Figure 3:**
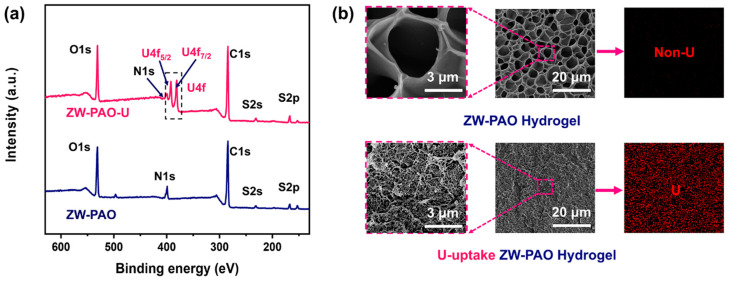
The characterization of the ZW-PAO hydrogel before and after U-extraction: (**a**) the XPS spectra; (**b**) the SEM and EDS mapping images.

The U-adsorption properties of the ZW-PAO hydrogel were qualitatively studied within a pure water solution containing 16 ppm U for 48 h. The U-uptake of the ZW-PAO hydrogel can be characterized by the XPS (Figure 3a and Figure S2). Compared with the original hydrogels, the hydrogels after U-extraction showed stronger U4f5/2 and U4f7/2 double peaks with bond energies of 392.7 eV and 381.8 eV, respectively. In addition, the SEM image also showed a clear difference after U-extraction. As shown in Figure 3b and Figure S3, owing to the further cross-linking of the ZW-PAO hydrogel after U-adsorption (based on the coordination between the adsorbed UO_2_^2+^ and the PAO) [24], the pore structure of the hydrogel was significantly denser than that before U-extraction, which can also be verified based on the EDS mapping. In addition, the U-adsorption of the ZW-PAO hydrogel can be further confirmed based on the comparison of the FT-IR adsorption spectroscopy (Figure S4) or wide-angle X-ray diffraction (Figure S5) between the original ZW-PAO hydrogel and the U-uptake of the ZW-PAO hydrogel [34,35,36]. More importantly, a series of hydrogels with different proportions of DVBAP:PAO were prepared (Table S2), whose U-adsorbing performance was analyzed and compared within the seawater with a 16 ppm U-concentration for 24 h. The U-adsorbing capacity gradually increased with the mass ratio increase in DVBAP: PAO from 5:0 to 5:2, owing to the increased PAO content of the ZW-PAO hydrogel (Figure S6). However, with the further increase in DVBAP:PAO from 5:2 to 5:5, the U-adsorption capacity was almost unchanged, which may be due to the decrease in hydrophilicity by the added relatively hydrophobic PAO in the hydrogel. Therefore, the ZW-PAO hydrogel with 5:2 was selected as the optimal ratio to fabricate the ZW-PAO hydrogel for following experiments.

### 2.4. Adsorption Kinetics of U-Adsorption Properties of ZW-PAO Hydrogel

**Figure 4:**
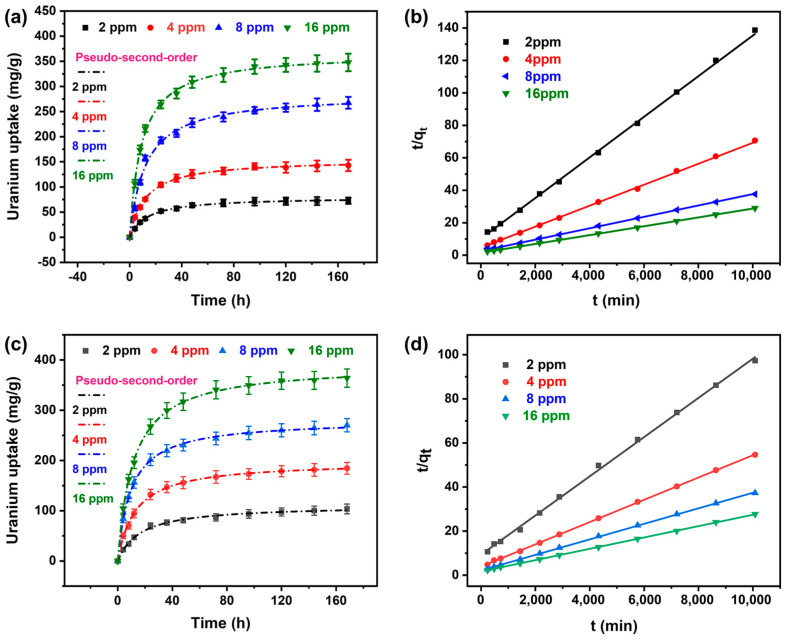
The U-adsorption kinetics of the ZW-PAO hydrogel and the pseudo-second-order in 2, 4, 8, and 16 ppm U-added (**a**,**b**) pure water and (**c**,**d**) seawater. All the solutions were adjusted to pH = 6 prior to U-adsorption.

The adsorption kinetics of U-adsorption performances of this ZW-PAO hydrogel were systemically researched (Figure 4). An adsorption isotherm experiment was applied to evaluate the U-adsorbing process of this ZW-PAO hydrogel (Figures S7–S9). Based on the Freundlich model, the Langmuir model fitted the real U-adsorbing isothermal kinetics well, which confirmed that the U-adsorption of this ZW-PAO hydrogel was single-layer chemisorption [37]. In order to explore whether the swelling property of the ZW-PAO hydrogel in seawater is beneficial to the U-adsorption process, we further investigated the adsorption kinetics of the U-adsorption properties the ZW-PAO hydrogel. The adsorption kinetic curves of the ZW-PAO hydrogel were obtained in pure water with U-concentrations of 2, 4, 8, and 16 ppm (Figure 4a); the adsorbing capacities reached 52.1 ± 3.6, 104.2 ± 7.9, 192.7 ± 12.4, and 264.1 ± 15.7 mg/g after 24 h, respectively. After the adsorption equilibrium, the adsorption capacities were 72.7 ± 6.4, 142.9 ± 11.3, 267.4 ±11.8, and 347.6 ±17.4 mg/g, respectively. As a contrast, the U-adsorbing capacity of the ZW-PAO hydrogel was measured in seawater solution (Figure 4c), and reached 69.8 ± 6.0, 132.3 ± 10.5, 201.6 ± 11.6, and 267.2 ± 15.1 mg/g after 24 h within 2, 4, 8, and 16 ppm of U-concentration, respectively. After the adsorption equilibrium, the U-adsorption amounts were 103.6 ± 9.7, 184.5 ± 11.8, 270.2 ± 13.2, and 363.9 ± 18.1 mg/g, respectively. In addition, the adsorption kinetic curves of the ZW-PAO hydrogels were fitted with the quasi-second-order kinetic models (Figure 4b,d, Table S3), proving that the U-adsorbing process of the ZW-PAO hydrogel belonged to chemisorption.

### 2.5. pH Dependency of U-Adsorbing Property and Ion Selectivity

**Figure 5:**
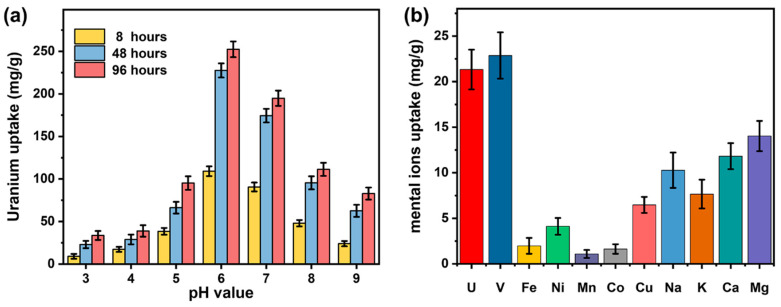
(**a**) U-extraction capacity as a function of pH in pure water containing 8 ppm U; (**b**) selective adsorption of metal ions by the ZW-PAO hydrogel.

The form of U can change at different pHs, which can affect the adsorbing capacity of adsorbents. The pH dependency of the U-adsorbing property was measured (Figure 5a). The U-adsorbing capacity of the ZW-PAO hydrogel with a change in pH in pure water with 8 ppm U was investigated. With the increase in pH from 3 to 6, the U-adsorbing capacity increased and reached a maximum at pH = 6, and then decreased gradually with the further increase in pH from 6 to 9. Diverse competitive ions exist in real seawater environments, which can significantly disturb the U-adsorbing capacity of the ZW-PAO hydrogel. Therefore, an experiment was designed to evaluate the U-adsorption performance of the ZW-PAO hydrogel in the presence of interfering elements. A mixture solution was prepared with 100 times the concentrations of U(VI), V(V), Fe^3+^, Ni^2+^, Mn^2+^, Co^2+^, and Cu^2+^ as in real seawater and concentrations of Na^+^, K^+^, Ca^2+^, and Mg^2+^ set invariability as in natural seawater (Table S4). As shown in Figure 5b, the U-adsorption amount was 21.33 ± 2.2 mg/g after 48 h, which was much higher than for other metal ions except for vanadium (V). Vanadium is the biggest competitor for the selective U-adsorption of the ZW-PAO hydrogel, due to the fact that the amidoxime group can form more stable coordination bonds with vanadium ions than with UO_2_^2+^ [38]. Therefore, this ZW-PAO hydrogel can be utilized to highly efficiently extract U from not only acidic uranium-containing waste freshwater but also alkaline uranium-containing natural seawater.

### 2.6. U-Adsorption–Desorption Recyclability of ZW-PAO Hydrogel

**Figure 6:**
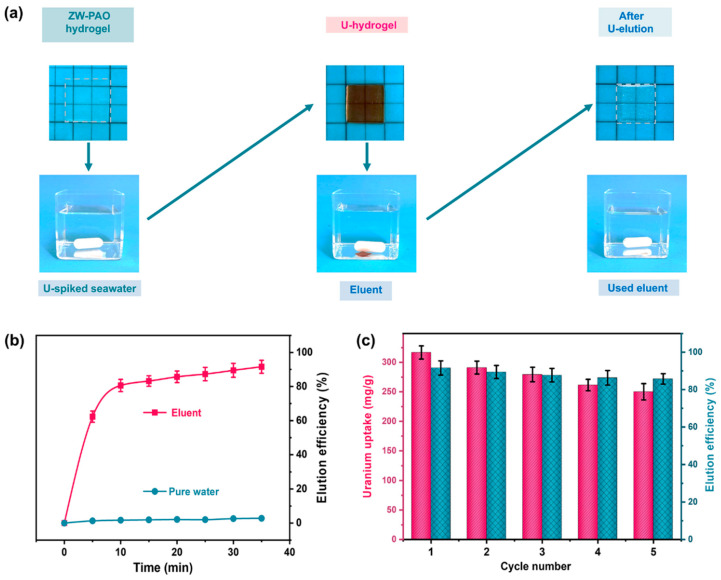
(**a**) U-adsorbing–desorbing process, (**b**) U-desorption kinetic curve and (**c**) 5 adsorbing–desorbing cycles of the ZW-PAO hydrogel.

In order to verify the recyclability of U-adsorption by the ZW-PAO hydrogel, a cyclic adsorption–desorption experiment was conducted (Figure 6). After 10 mg of adsorbent was adsorbed in seawater with 16 ppm U for 48 h, rapid desorption of the hydrogel in eluent took place for 35 min. It is interesting that, after elution, the ZW-PAO hydrogel recovered from reddish to transparent (Figure 6a), which indicated the high desorbing rate. The desorbing rate of the first desorption reached 91.6 ± 3.8% (Figure 6b). The ZW-PAO hydrogel was subjected to five cyclic adsorption–desorption experiments (Figure 6c). Compared with the initial adsorption capacity of the hydrogel at 316.8 ± 11.2 mg/g, the regenerated hydrogel could maintain 251.2 ± 13.8 mg/g of U-adsorbing capacity, and the desorbing ratio could maintain 85.7 ± 2.8% after five cycles, which indicated that the ZW-PAO hydrogel had excellent U-adsorption reusability.

### 2.7. Anti-Biofouling Properties of ZW-PAO Hydrogel

**Figure 7:**
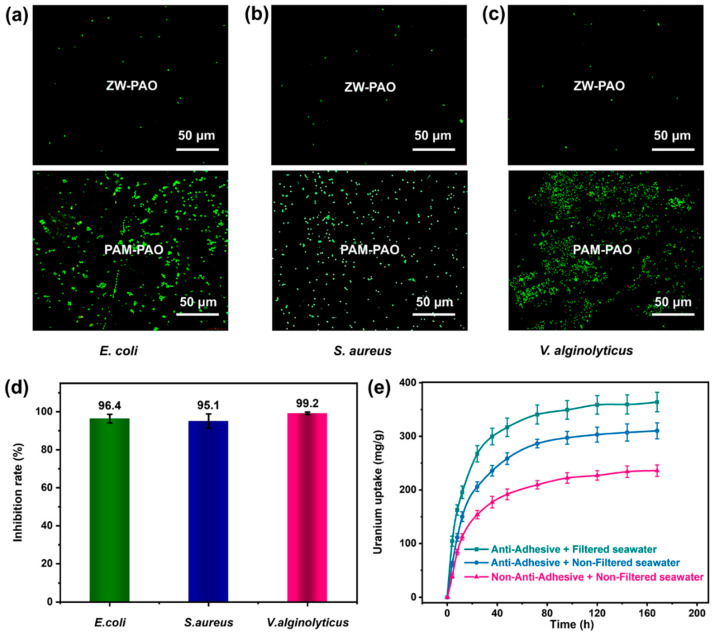
(**a**–**c**) The confocal laser microscopy of different ZW-PAO and PAM-PAO hydrogels; (**d**) the antibacterial rates of the ZW-PAO hydrogel against three types of bacteria; (**e**) the enhanced U-adsorption capacity of the ZW-PAO hydrogel by the improved anti-biofouling in seawater.

The anti-biofouling of the ZW-PAO hydrogel was tested by using *Escherichia coli*, *Staphylococcus aureus*, and *Vibrio alginolyticus*. Firstly, both the ZW-PAO and PAM-PAO hydrogels were attached with a large number of bacteria on their surfaces. Then, they were oscillated in the filtered seawater solution for 10 min to remove the bacteria on the surface. According to the confocal laser microscopy in Figure 7a–c, only a few bacteria can adhere onto the ZW-PAO hydrogel, which indicates outstanding anti-biofouling properties. On the contrary, a great deal of bacteria still adhered onto the blank PAM-PAO hydrogel. The antibacterial rates of the ZW-PAO hydrogel against these three types of bacteria were 96.4 ± 2.29%, 95.1 ± 3.76%, and 99.2 ± 0.63%, respectively (Figure 7d), which indicated that the anti-biofouling performance of the ZW-PAO hydrogel was better than that of the PAM-PAO hydrogel in filtered seawater. The uranium adsorption performance of ZW-PAO can be improved by anti-biofouling (Figure 7e): ZW-PAO and PAM-PAO without anti-biofouling properties were put into U-added seawater, which was divided into filtered and without filtering bacteria/microorganisms. Bacteria and microorganisms would seriously interfere with the adsorption capacity of ZW-PAO for uranium, and it was seen that the adsorption capacity of ZW-PAO and PAM-PAO for uranium in natural seawater containing bacteria and microorganisms was substantially lower for both than the uranium adsorption of ZW-PAO in filtered seawater without bacteria and microorganisms (363.9 ± 18.1 mg/g). Meanwhile, in natural seawater, compared with the uranium adsorption performance of PAM-PAO without anti-biofouling, the uranium adsorption of ZW-PAO was dramatically increased by 31.9% from 235.8 ± 10.7 mg/g to 310.1 ± 14.9 mg/g. Therefore, the excellent anti-biofouling properties can substantially enhance the uranium adsorption capacity of the material, which can efficiently extract uranium from a natural seawater environment over a relatively long period of time. The mechanism of this excellent anti-biofouling performance of the ZW-PAO hydrogel can be explained by the anti-polyelectrolyte effect [32,33]: a large number of paired positive and negative charges of the hydrogel network can provide ion–dipole interactions with surrounding water molecules, forming a stable and condensed layer on the surface of the ZW-PAO hydrogel. Therefore, this ZW-PAO hydrogel demonstrated excellent anti-biofouling behavior, on account of this super-hydrophilic surface layer to prevent the adhesion of bacteria and microorganisms.

### 2.8. U-Extracting Ability of the ZW-PAO Hydrogel in Real Seawater

The above adsorption effect of adsorbents in a high-concentration U solution is often different from that in real seawater. In order to verify whether the ZW-PAO hydrogel has the prospect of large-scale U-extraction in real seawater without filtering bacteria, the long-term adsorbing capacity of the ZW-PAO hydrogel was tested using natural seawater with and without filtering bacteria. Meanwhile, the PAM-PAO hydrogel was used as a control to compare the anti-biofouling effect of the ZW-PAO hydrogel on the U-extraction performance in real seawater. The sea test experiments were carried out with 10 mg dry weight adsorbent in 100 L natural seawater without filtering bacteria. Seawater samples were taken every 5 days, to test the difference in U-concentration in seawater before and after adsorption to calculate the U-adsorbing capacity of the hydrogels.

**Figure 8:**
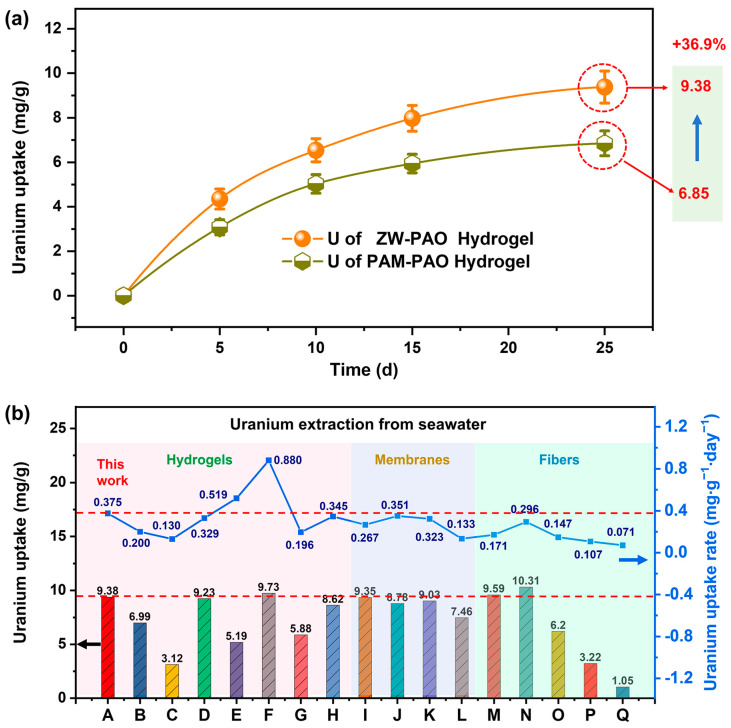
(**a**) Comparison of real seawater adsorption kinetics between ZW-PAO and PAM-PAO hydrogels; (**b**) comparison of uranium extraction rate between ZW-PAO hydrogel and existing amidoxime-group adsorbents [20,21,39,40,41,42,43,44,45,46,47,48,49,50,51,52].

The U-adsorbing capacity of the ZW-PAO hydrogel reached 9.38 ± 0.72 mg/g after 25 d in natural seawater without filtering bacteria, which was 36.9% higher than that of the blank PAM-PAO hydrogel (Figure 8a). This further proved that the anti-biofouling effect of the ZW-PAO hydrogel significantly improved the ability of U-extraction. In addition, the average adsorption rate of the ZW-PAO hydrogel was calculated as 0.375 mg/(g∙day). Finally, transverse comparison with the current hydrogel, membrane, and fiber U-adsorbents with the amidoxime group are shown in Figure 8b and Table S5. The ZW-PAO hydrogel had a high adsorption capacity and adsorption rate, superior to most existing uranium adsorbents. Therefore, the ZW-PAO hydrogel could be a promising U-adsorbent and will have application prospects in large-scale U-extraction from seawater in real ocean environments. In addition, this ZW-PAO hydrogel could also have potential applications in U-recovery from contaminated uranium-containing waste seawater.

## 3. Conclusions

In summary, we have developed a ZW-PAO hydrogel adsorbent with both high-efficiency U-extraction and biological fouling resistance in seawater. This adsorbent not only enhances the hydrophilicity of the hydrogel in seawater through the inherent “anti-polyelectrolyte effect” of zwitterions, but also forms a dense hydration layer on the surface of the hydrogel through ion–dipole interaction, which makes it have excellent resistance to the adhesion of microorganisms, so that it can maintain high-efficiency uranium extraction performance in seawater for a long time. The U-adsorbing capacity of the ZW-PAO hydrogel reached 9.38 ± 0.72 mg/g at 25 days, which was 36.9% higher than the PAM-PAO hydrogel, which is excellent in the existing adsorbents for U-extraction from seawater. This research has important scientific significance and broad application prospects. This ZW-PAO hydrogel will be a promising adsorbent for large-scale U-recovery from real seawater and will inspire the development of hydrogel-based and other types of U-adsorbents. 

## 4. Materials and Methods

### 4.1. Materials

Polyacrylonitrile (PAN, Mw ≈ 150K, 97.0%), 3-(dimethyl 4-vinylbenzyl amino) propyl sulfonate (DVBAP, 98.0%), acrylamide (AM, 97.0%), N,N′-methylene bisacrylamide (BIS, 99.0%), photoinitiator I-2959 (99.5%), hydroxylamine hydrochloride (NH_2_OH·HCl, 97.5%), N,N-dimethylformamide (DMF, 99.0%), sodium carbonate (Na_2_CO_3_, 99.5%), sodium chloride (NaCl, 99.5%), sodium hydroxide (NaOH, 99.5%), sodium bicarbonate (NaHCO_3_, 99.7%) Arsine azo (III) (99.7%), hydrogen peroxide (H_2_O_2_, 98.5%), ethanol (99.5%), and potassium carbonate (K_2_CO_3_, 99.5%) were bought from Xilong Scientific Co., Ltd. (Shantou, China)The uranyl nitrate hexahydrate [UO_2_(NO_3_)_2_·6H_2_O] was purchased from Qifei Pharmaceutical Chemical Co., Ltd (Tianmen, China). All the materials above were used directly without further purification. The seawater used for the tests was taken from the sea area of Wenchang City, Hainan Province.

### 4.2. Characterization

Fourier transform infrared spectroscopy (FT-IR) was performed with a Nicolet 7199 spectrometer (Thermo Fisher Scientific, Madison, WI, USA). X-ray photoelectron spectroscopy (XPS) measurements were obtained with a Thermo Scientific (Waltham, MA, USA)Escalab 250 Xi spectrometer. Morphologies were researched via transmission electron microscopy (JOEL, JEM 2100 Tokyo, Japan) and scanning electron microscopy (Hitachi, S-4800 Ibaraki Prefecture Japan). The pH values of the U-added simulated seawater were detected via a pH meter (F2, Mettler Toledo Zurich, Switzerland). Ultraviolet–Visible (UV–Vis) spectra were obtained with a spectrophotometer (UV 1800 PC, AuCy Instrument, Shanghai, China). The adsorption capacity of the hydrogel in pure water and real seawater was measured by ICP-OES (iCAP 7600, Thermo Scientific, MA, USA). The contact angle tester was made by (PZ-80SD) Beijing Pinzhi Chuangsi. Comparison of the wide-angle X-ray diffraction (WAXD) (Brooke D8 Karlsruhe, Germany) between original PZW-PAO hydrogel and U-uptake PZW-PAO hydrogel.

### 4.3. Experimental Methods

#### 4.3.1. Preparation of PAO

The PAO was prepared according to our reported method [24] (Figure S10a). Firstly, NaOH (1.60 g), Na_2_CO_3_ (4.12 g), and NH_2_OH·HCl (5.60 g) were dissolved in DMF (60 mL). After stirring at 45 °C for 1 h, PAN (4.00 g) was slowly added. The temperature was then increased to 65 °C for 24 h. After filtering, the supernatant was slowly dropped into ethanol to obtain a white flocculent precipitation. Finally, after filtration, the sediment was dried overnight at 60 °C and subsequently was ground into a fine powder to obtain the as-prepared PAO product.

#### 4.3.2. Preparation of ZW-PAO Hydrogel

The synthesis of the semi-interpenetrating ZW-PAO hydrogel is illustrated in Figure S10b. A typical procedure for the preparation of the ZW-PAO hydrogel with the mass ratio of DVBAP:PAO = 5:2 was carried out as follows: the PAO powder (40 mg) was dissolved in sodium hydroxide solution (0.3 mol/L, 0.6 mL), and then the DVBAP monomer (100 mg), I-2959 photoinitiator (1 mg), and BIS crosslinker (1 mg) were added to obtain the precursor solution after mixing. The precursor solution was transferred to a mold made of silicone rubber and quartz glass of different thicknesses and photopolymerized for 2 h to obtain the semi-interpenetrating ZW-PAO hydrogel. A polyacrylamide–poly(amidoxime) (PAM-PAO) blank hydrogel was prepared as the control group. The specific formula was PAO powder (40 mg) dissolved in sodium hydroxide solution (0.3 mol/L, 0.6 mL), followed by the AM monomer (100 mg), AIBA photoinitiator (4 mg), and BIS crosslinker (4 mg). After mixing, the precursor liquid was transferred to the mold for photopolymerization for 10 min to obtain the PAM-PAO hydrogel. The hydrogel was purified by immersing in 500 mL deionized water which was changed every 8 h. After 48 h of purification, the hydrogel was stored in deionized water at 0–5 °C before use. Furthermore, the dry gel mass can be calculated based on the constant mass percentage content (wt%) of the dry gel in the ZW-PAO hydrogel (Table S1). Therefore, the dry mass of one hydrogel sample can be obtained conveniently, through weighing the mass of the wet hydrogel without the need to dry it.

#### 4.3.3. Adsorption Performance of ZW-PAO Hydrogel

Different concentrations of U-solution in pure water and seawater were prepared. The U-concentration of U-spiked solutions can be tested based on the Arsine azo (III) method through a UV–visible spectrophotometer. The standard regression curves of the absorbance of different U-concentrations in pure water (Figure S11) and seawater (Figure S12) were calculated, respectively. The U-adsorbing kinetics were studied by immersing the adsorbent (with 5 mg of dry gel) into 1 L of U-solution at pH = 6. During the adsorption process, the adsorbent and the U-addition solution were continuously shaken by a shaker at room temperature to simulate the flow of seawater. To evaluate the adsorbing type, the adsorbing kinetics of hydrogels were calculated based on both the quasi-first-order kinetic model and quasi-second-order kinetic model.

#### 4.3.4. Ion-Selective Adsorption Performance of ZW-PAO Hydrogel

When various metal ions (UO_2_^2+^, VO^3+^, Fe^3+^, Ni^2+^, Mn^2+^, Co^2+^, Cu^2+^) were added to real seawater, the concentrations of these elements above were 100 times those of natural seawater, but the concentrations of Na^+^, K^+^, Ca^2+^, Mg^2+^ were consistent with those of real seawater. After the semi-interpenetrating ZW-PAO hydrogel adsorbents (10 mg) were immersed into the solution (1 L) for 48 h with oscillation at 25 °C, each ion concentration before and after adsorption was analyzed by ICP-MS to evaluate the adsorption capacity of this hydrogel adsorbent for each ion.

#### 4.3.5. U-Adsorption–Desorption Cyclic Test

Firstly, a ZW-PAO hydrogel with 10 mg of dry gel was immersed in 1 L of 16 ppm U-uptake seawater (pH = 6.0) for 24 h at room temperature to extract U. Then, the U-uptake ZW-PAO hydrogel can be easily desorbed with an elution with Na_2_CO_3_ (1.0 M) and H_2_O_2_ (0.1 M). This U-uptake adsorbent was placed in 200 mL eluent for 35 min to desorb U from the hydrogel. It is worth noting that the completely eluted hydrogel should be washed with ultra-pure water several times before the next adsorption until the washing liquid is pH = 7; otherwise, the adsorption effect of the adsorbent will be affected. Cyclic U-adsorption was conducted within 1.0 L of U-added (16 ppm) seawater, which is the same condition as the first U-adsorption. The formula for calculating elution efficiency was as follows:

$$Elution\;rate=\frac{C_{el}\times V_{el}}{W_{e}}$$

where “*C_el_*” (mg/L) is the eluent U-concentration, “*V_el_*” (L) is the eluent volume, and “*W_e_*” (mg) is the dry mass of the adsorbent after the U-adsorption.

#### 4.3.6. The Anti-Bioadhesion Properties of Hydrogel Adsorbents

*Escherichia coli*, *Staphylococcus aureus*, and *Vibrio alginolyticus* were selected for bacterial staining experiment. The sample used a disk (1 cm of diameter and 0.5 mm of thickness). Both the ZW-PAO hydrogel and PAM-PAO hydrogel were soaked in 75% ethanol for 30 min, and then washed and dried with PBS for reserve use. The sterilized disk was placed in a 24-well plate, the bacterial solution was diluted to 108 CFU/mL with PBS, 2.0 mL of the diluted bacterial solution was poured into the hole, and then the bacteria could be cultured at 37 °C, 200 rpm for 6 h. After completion, the samples were put into the filtered seawater solution and placed on the shaking table at 100 rpm for 10 min. The dyeing solution was obtained by adding SYTO-9 (2 μL) and PI (2 μL) into 2 mL PBS solution. Finally, 200 μL of dyeing liquid was added to the sample and stained at 37 °C for 15 min without light. After staining, the samples were placed on a confocal laser microscope to observe the bacterial adhesion with an oil lens at 600 times magnification.

#### 4.3.7. U-Adsorbing Capacity of ZW-PAO Hydrogel in Real Seawater

According to the reported literature [24], a real seawater (without filtering bacteria) adsorption system was designed, in which a dry hydrogel (10 mg) was fixed between two sponges and transferred to an adsorption column. Each adsorption column was equipped with 100 L of natural seawater, and the flow rate was stabilized at 90 L/h. The real seawater U-adsorbing process was conducted for 25 days, and samples were taken every 5 days to test the U-adsorbing capacity.

#### 4.3.8. Preparation of Different Concentrations of Uranium Solution

First, uranyl nitrate hexahydrate (2.1092 g) was dissolved in 500 mL of ultra-pure water, then transferred to a 1 L volumetric bottle and ultra-pure water was used to set the volume, and finally a 1000 ppm uranium standard solution was obtained. According to the standard procedure proposed by Oak Ridge National Laboratory, the simulated seawater was prepared by adding NaCl (25.6 g/L) and NaHCO_3_ (0.193 g/L) in ultra-pure water. Then, the simulated seawater was used to dilute an over 1000 ppm uranium standard solution to obtain different concentrations of uranium. In order to study the effect of pH value on the adsorption performance of the adsorbent, simulated seawater solutions of uranium with different pH values were prepared by using HCl (0.1 mol/L) or NaOH (0.1 mol/L) solution.

#### 4.3.9. Uranium Adsorption Performance Test

The Arsine azo (III) method was used to determine the concentration of uranium in pure water and simulated seawater. Arsine azo III is a color developing agent; it can react quickly with uranyl ions to form a blue chelate, of which the maximum absorption wavelength is 652 nm. Uranyl solution of different concentrations (0.5 mL), ultrapure water (3 mL), Arsine azo (III) (1 mL, 500 mg/L), and HCl (0.5 mL, 0.1 mol/L) were mixed to obtain the solution for detection. The absorbance at 652 nm was determined by a UV–visible spectrophotometer; the functional relationship between uranyl ion concentration and absorbance can be studied. Therefore, preparing the standard curve of uranium concentration prediction in advance can easily and accurately measure the uranium concentration of the solution to be measured by a UV–vis spectrophotometer. Take the pure water and simulated seawater standard solutions with different uranium concentrations from 0 ppm to 40 ppm (with an interval of 4 ppm) and add 0.5 mL of each solution to the mixed liquid (4.5 mL) prepared according to the above ratio and measure the absorbance of each mixed solution. After linear fitting, standard regression curves of different uranium concentration-absorbance can be obtained.

A series of pure water and simulated seawater with different uranium concentrations (2 ppm, 4 ppm, 8 ppm, and 16 ppm) was prepared. The adsorption kinetics were studied by adding adsorbent (with 5 mg of dry gel) into 1 L uranium solution with pH = 6. During the adsorption process, the adsorbent and the uranium addition solution were continuously shaken by a shaker at room temperature to simulate the flow of seawater. A liquid sample (2 mL) was removed from the solution at appropriate intervals for analysis. Uranium adsorption capacity is calculated according to Formula (1):

(1) $$q_{t}=\left(C_{o}-C_{t}\right)\times \frac{V}{m}$$

where “*q_t_*”(mg-U/g-Ads) is the adsorption amount of uranium at a specific time (t); “*C*_0_” and “*C_t_*” (mg/L) are uranium concentrations in pure water or simulated seawater at the initial time and a specific time, respectively. “*V*” is the volume of pure water or simulated seawater (L) and “*m*” is the mass of the adsorbent (g).

#### 4.3.10. Calculation Method of Adsorption Kinetics/Thermodynamics

In order to evaluate the adsorption type of adsorbent, the adsorption kinetics of the hydrogel were analyzed by using a quasi-first-order kinetic model (Formula (2)) and quasi-second-order kinetic model (Formula (3)).

(2) $$\ln(q_{e}-q_{t})=\ln q_{e}-k_{1}t$$

(3) $$\frac{t}{q_{t}}=\frac{1}{k_{2}{q_{e}}^{2}}+\frac{t}{q_{e}}$$

In the formula, “*q_e_*” (MG-U/G-ADS) is the theoretical adsorption capacity of uranium when the adsorbent reaches the adsorption equilibrium state, “*t*” is the adsorption time (min), and “*k*_1_” (Min^−1^) and “*k*_2_” [g/(mg∙min)] are the quasi-first-order and quasi-second-order adsorption rate constants, respectively. If the adsorption kinetic curve is more consistent with the quasi-second-order kinetic model, it proves that the adsorption of uranium by the material belongs to chemical adsorption; if the fitting effect is better with the quasi-first-order kinetic model, it proves that the adsorption of uranium by the material belongs to physical adsorption.

In order to test the saturation adsorption capacity of the adsorbent, the adsorption behavior of uranium with different concentrations (2, 4, 8, 16, 32, 64, 128 ppm) was evaluated by an isothermal adsorption experiment at 25 °C. The equilibrium isothermal data were fitted by Langmuir (Formula (4)) and Freundlich (Formula (5)) models, respectively.

(4) $$\frac{C_{e}}{q_{e}}=\frac{C_{e}}{q_{m}}+\frac{1}{q_{m}K_{L}}$$

(5) $$lnq_{e}=lnK_{F}+\frac{1}{n}lnC_{e}$$

where “*C_e_*” (mg/L) and “*q_e_*” (mg/g) are the concentration and adsorption amount of the hydrogel at equilibrium. “*q_m_*” is the saturated adsorption capacity (mg/g). “*K_L_*” is the equilibrium constant (L/mg) of the binding strength of the adsorbent and uranyl ion. “*K_F_*” is the Freundlich constant and “*n*” is the Freundlich index.

Different concentrations of uranium solution in pure water and simulated seawater were prepared. The Arsine azo (III) method was used to determine the concentration of uranium by a UV–visible spectrophotometer. The standard regression curves of the absorbance of different uranium concentrations in pure water and simulated seawater were obtained (Figures S10 and S11). The adsorption kinetics were studied by adding adsorbent (with 5 mg of dry gel) into 1 L uranium solution with pH = 6. During the adsorption process, the adsorbent and the uranium addition solution were continuously shaken by a shaker at room temperature to simulate the flow of seawater. In order to evaluate the adsorption type of adsorbent, the adsorption kinetics of hydrogel were analyzed by using a quasi-first-order kinetic model and quasi-second-order kinetic model.

## Data Availability

Data are contained within the article and Supplementary Materials.

## Supplementary Materials

The following supporting information can be downloaded at: https://www.mdpi.com/article/10.3390/gels10090603/s1, Figure S1. Comparison of the air/seawater contact angle change in the PZW-PAO hydrogel and the blank PAM-PAO hydrogel; Figure S2. The split peak analysis of the U-uptake ZW-PAO hydrogel; Figure S3. The EDS mapping analysis of the U-uptake PZW-PAO hydrogel; Figure S4. Comparison of FT-IR spectroscopy between the before and after U-uptake PZW-PAO hydrogel; Figure S5. Comparison of the wide-angle X-ray diffraction (WAXD) between the before and after U-uptake PZW-PAO hydrogel. Figure S6. Uranium adsorption properties of hydrogels with different ratios (m_DVBAP_: m_PAO_ = 5:0 to 5:5); Figure S7. The adsorption thermodynamic curve of the PAO-PZW hydrogel and Langmuir model and Freundlich model; Figure S8. The linear Langmuir isotherm model for the U-adsorption; Figure S9. The linear Freundlich isotherm model for the U-adsorption; Figure S10. (a) Preparation of the PAO polymer. (b) The illustration on the fabrication and semi-interpenetrating structure of the ZW-PAO hydrogel; Figure S11. Standard curve for prediction of uranium concentration in pure water; Figure S12. Standard curve for prediction of uranium concentration in seawater; Table S1. Comparison of swelling ratio and water content of ZW-PAO hydrogel in pure water and seawater. Table S2. PAO contents in different precursor solutions of ZW-PAO hydrogels; Table S3. The pseudo-second-order model for the uranium adsorption kinetics of the ZW-PAO hydrogel in 2, 4, 8, 16 ppm uranium-added seawater; Table S4. Concentration of U(VI) and co-existing metal ions in seawater and ×100 seawater. Table S5. Comparison of uranium extraction rate between ZW-PAO hydrogel and existing amidoxime group adsorbent.

## References

[1] Kaltsoyannis N., Liddle S.T. (2016). Catalyst: Nuclear power in the 21st century. Chem.

[2] Dungan K., Butler G., Livens F.R., Warren L.M. (2017). Uranium from seawater–Infinite resource or improbable aspiration?. Prog. Nucl. Energy.

[3] Liu S.B., Wang Y.Z., Tang Y.F., Fu X.Z., Luo J.L. (2024). Emerging Nanomaterials toward Uranium Extraction from Seawater: Recent Advances and Perspectives. Small.

[4] Li P., Zhun B., Wang X., Liao P.P., Wang G.H., Wang L.Z., Guo Y.D., Zhang W.M. (2017). Highly Efficient Interception and Precipitation of Uranium(VI) from Aqueous Solution by Iron-Electrocoagulation Combined with Cooperative Chelation by Organic Ligands. Environ. Sci. Technol..

[5] Hernández J., Ruiz D. (2021). Removal of chloride ions from a copper leaching solution, using electrodialysis, to improve the uranium extraction through ion-exchange. J. Hazard. Mater..

[6] Chu J., Huang Q.G., Dong Y.H., Yao Z.E., Wang J.R., Qin Z., Ning Z.G., Xie J.J., Tian W., Yao H.J. (2022). Enrichment of uranium in seawater by glycine cross-linked graphene oxide membrane. Chem. Eng. J..

[7] Wang J.J., Wang Y., Wang W., Ding Z., Geng R.Y., Li P., Pan D.Q., Liang J.J., Qin H.B., Fan Q.H. (2020). Tunable mesoporous g-C_3_N_4_ nanosheets as a metal-free catalyst for enhanced visible-light-driven photocatalytic reduction of U (VI). Chem. Eng. J..

[8] Abney C.W., Mayes R.T., Saito T., Dai S. (2017). Materials for the recovery of uranium from seawater. Chem. Rev..

[9] Wu Y., Xie Y.H., Liu X.L., Li Y., Wang J.Y., Chen Z.S., Yang H., Hu B., Shen C., Tang Z.W. (2023). Functional nanomaterials for selective uranium recovery from seawater: Material design, extraction properties and mechanisms. Coord. Chem. Rev..

[10] Endrizzi F., Rao L.F. (2014). Chemical speciation of uranium (VI) in marine environments: Complexation of calcium and magnesium ions with [(UO_2_) (CO_3_)_3_]^4−^ and the effect on the extraction of uranium from seawater. Chem.-Eur. J..

[11] Maity S., Sahu S.K., Pandit G.G. (2015). Standardization of solvent extraction procedure for determination of uranium in seawater. J. Radioanal. Nucl. Chem..

[12] Ma D.S., Xu X., Li Z.W., Peng H., Cai D., Wang D., Yue Q. (2022). Nanoemulsion assembly toward vaterite mesoporous CaCO_3_ for high-efficient uranium extraction from seawater. J. Hazard. Mater..

[13] Hu E.M., Chen Q., Gao Q., Fan X.F., Luo X.J., Wei Y., Wu G., Deng H.B., Xu S.C., Wang P. (2024). Cyano-Functionalized Graphitic Carbon Nitride with Adsorption and Photoreduction Isosite Achieving Efficient Uranium Extraction from Seawater. Adv. Funct. Mater..

[14] Chatterjee S., Bryantsev V.S., Brown S., Johnson J.C., Grant C.D., Mayes R.T., Hay B.P., Dai S., Saito T. (2016). Synthesis of naphthalimidedioxime ligand-containing fibers for uranium adsorption from seawater. Ind. Eng. Chem. Res..

[15] He J.R., Sun F.L., Han F.H., Gu J.J., Ou M.R., Xu W.K., Xu X.P. (2018). Preparation of a novel polyacrylic acid and chitosan interpenetrating network hydrogel for removal of U (VI) from aqueous solutions. RSC Adv..

[16] He J., Jin J., Wang Z.Z., Yin H.W., Wei C.C., Xu X.P. (2018). Encapsulating nanosilica into polyacrylic acid and chitosan inter-penetrating network hydrogel for preconcentration of uranium from aqueous solutions. J. Radioanal. Nucl. Chem..

[17] Song Y.C., Tan H.H., Qin S.L., Liu Z., Liu C.T., Shen C.Y., Yang P.P., Li S.W. (2024). Assembly of a core-shell MOF with stability into Polyacrylamide hydrogel for boosting extraction of uranium from seawater. Nano Res..

[18] Ye H., Liu C., Wu M.B., Ma L.L., Liu S.C., Zhong Y., Yao J.M. (2022). Amyloid-like assembly converting commercial proteins to water-insoluble adsorbents with ultrahigh adsorption capacity and excellent antifouling property for uranium extraction. J. Mater. Chem. A.

[19] Tang N., Liang J., Niu C.G., Wang H., Luo Y., Xing W.L., Ye S.J., Liang C., Guo H., Guo J.Y. (2020). Amidoxime-based materials for uranium recovery and removal. J. Mater. Chem. A.

[20] Li Z., Yu Z.Q., Wu Y.D., Wu X.L., Wan Y., Yuan Y.H., Wang N. (2020). Self-sterilizing diblock polycation-enhanced polyami-doxime shape-stable blow-spun nanofibers for high-performance uranium capture from seawater. Chem. Eng. J..

[21] Shi S., Qian Y.X., Mei P.P., Yuan Y.H., Jia N., Dong M.Y., Fan J.C., Guo Z.H., Wang N. (2020). Robust flexible poly (amidoxime) porous network membranes for highly efficient uranium extraction from seawater. Nano Energy.

[22] Hao M.J., Xie Y.H., Liu X.L., Chen Z.S., Yang H., Waterhouse G., Ma S.Q., Wang X.K. (2023). Modulating uranium extraction performance of multivariate covalent organic frameworks through donor–acceptor linkers and amidoxime nanotraps. JACS Au.

[23] Li J., Tuo K., Fan C.B., Liu G., Pu S.Z., Li Z.J. (2024). Hierarchical porous amidoximated metal–organic framework for highly efficient uranium extraction. Small.

[24] Ma C.X., Gao J.X., Wang D., Yuan Y.H., Wen J., Yan B.J., Zhao S.L., Zhao X.M., Sun Y., Wang X.L. (2019). Sunlight polymerization of poly(amidoxime) hydrogel membrane for enhanced uranium extraction from seawater. Adv. Sci..

[25] Sahu B., Pattnayak B., Mohapatra S. (2024). Halfa grass assembled double-layered hydrogel evaporator for sunlight-driven steam generation and selective uranium extraction. Desalination.

[26] Park J.Y., Gill G., Strivens J.E., Kuo L.J., Jeters R.T., Avila A., Wood J.R., Schlafer N.J., Janke C.J., Miller E. (2016). Effect of biofouling on the performance of amidoxime-based polymeric uranium adsorbents. Ind. Eng. Chem. Res..

[27] Li S., Feng K., Li J.Y., Li Y., Li Z.T., Yu L.M., Xu X.T. (2024). Marine antifouling strategies: Emerging opportunities for seawater resource utilization. Chem. Eng. J..

[28] Li H., He N.N., Cheng C., Dong H., Wen J., Wang X.L. (2020). Antimicrobial polymer contained adsorbent: A promising candidate with remarkable anti-biofouling ability and durability for enhanced uranium extraction from seawater. Chem. Eng. J..

[29] Li L., Li H., Lin M.Z., Hu S., Wen J. (2024). Zwitterionic functionalized chitosan with dual-antifouling for selective uranium ex-traction. Sep. Purif. Technol..

[30] Jiao G.J., Ma J.L., Zhang J.Q., Li Y.C., Liu K.N., Sun R.C. (2022). Porous and biofouling-resistant amidoxime-based hybrid hydrogel with excellent interfacial compatibility for high-performance recovery of uranium from seawater. Sep. Purif. Technol..

[31] Li H., Sun J., Qin S.L., Song Y.C., Liu Z., Yang P.P., Li S.W., Liu C.T., Shen C.Y. (2023). Zwitterion functionalized graphene oxide/polyacrylamide/polyacrylic acid hydrogels with photothermal conversion and antibacterial properties for highly efficient uranium extraction from seawater. Adv. Funct. Mater..

[32] Huang L., Zhang L.X., Xiao S.W., Yang Y., Chen F., Fan P., Zhao Z.P., Zhong M.Q., Yang J.T. (2018). Bacteria killing and release of salt-responsive, regenerative, double-layered polyzwitterionic brushes. Chem. Eng. J..

[33] Georgiev G.S., Kamenska E.B., Vassileva E.D., Kamenova I.P., Georgieva V.T., Iliev S.B., Ivanov I.A. (2006). Self-assembly, antipolyelectrolyte effect, and nonbiofouling properties of polyzwitterions. Biomacromolecules.

[34] Cao H., Bao H., Lin X., Lin J., Zhang L., Huang Y., Wang J.Q. (2019). Differential interplay between Ce and U on local structures of U1-xCexO2 solid solutions probed by X-ray absorption spectroscopy. J. Nucl. Mater.

[35] Wang W.G., Zhang S.L., Lu J. (1981). Infrared spectral characteristics of some common uranium minerals. Chin. J. Geol..

[36] Qiu L.F., Ou G.X., Zhang M., Li Q., Wu D., Shang C.J. (2016). Micro-area analysis of uranium minerals by Micro FT-IR spectrometry. Acta Mineral Sin..

[37] Wang J.W., Sun Y., Zhao X.M., Chen L., Peng S.Y., Ma C., Duan G.G., Liu Z.Z., Wang H., Yuan Y.H. (2022). A poly (amidoxime)-modified MOF macroporous membrane for high-efficient uranium extraction from seawater. e-Polymers.

[38] Ivanov A.S., Leggett C.J., Parker B.F., Zhang Z., Arnold J., Dai S., Abney C.W., Bryantsev V.S., Rao L. (2017). Origin of the unusually strong and selective binding of vanadium by polyamidoximes in seawater. Nat. Commun..

[39] Bai Z.Y., Liu Q., Zhang H.S., Yu J., Chen R.R., Liu J.Y., Song D.L., Li R.M., Wang J. (2020). Anti-biofouling and water—Stable balanced charged metal organic framework-based polyelectrolyte hydrogels for extracting uranium from seawater. ACS Appl. Mater. Interfaces.

[40] Zhang J.Q., Ma J.L., Jiao G.J., Liu K.N., Cui R., Zhai S.R., Sun R.C. (2023). Methyl 4-hydroxybenzoate nanospheres anchored on poly (amidoxime)/polyvinyl alcohol hydrogel network with excellent antibacterial activity for efficient uranium extraction from seawater. Desalination.

[41] Yan B.J., Ma C.X., Gao J.X., Yuan Y.H., Wang N. (2020). Anion-crosslinked supramolecular hydrogel for ultrahigh and fast ura-nium recovery from seawater. Adv. Mater..

[42] Zhang C.R., Cui W.R., Niu C.P., Yi S.M., Liang R.P., Qi J.X., Chen X.J., Jiang W., Zhang L., Qiu J.D. (2022). rGO-based covalent organic framework hydrogel for synergistically enhance uranium capture capacity through photothermal desalination. Chem. Eng. J..

[43] Liu T., Zhao J.T., Qiao Q.T., Zhang R.Q., Wei T., Liang Y.X., Yuan Y.H., Wang N. (2024). Engineering shrinkage resistance of nano-structured hydrogels in seawater for fast uranium capture. Chem. Eng. J..

[44] Liu R.R., Wen S.X., Sun Y., Yan B.J., Wang J.W., Chen L., Peng S.Y., Ma C., Cao X.Y., Ma C.X. (2021). A nanoclay enhanced Amidoxime-Functionalized Double-Network hydrogel for fast and massive uranium recovery from seawater. Chem. Eng. J..

[45] Song Y.C., Ma X., Tan H.H., Liu Z., Liu C.T., Shen C.Y., Yang P.P., Li S.W. (2023). Hollow Zn/Co zeolitic imidazolate framework-implanted composite hydrogel for highly efficient uranium extraction from seawater. Nano Res..

[46] Sun Y., Liu R.R., Wen S.X., Wang J.W., Chen L., Yan B.J., Peng S.Y., Ma C., Cao X.Y., Ma C.X. (2021). Antibi-ofouling ultrathin poly(amidoxime) membrane for enhanced U(VI) recovery from wastewater and seawater. ACS Appl. Mater. Interfaces.

[47] Yang L.S., Xiao H.Y., Zhao X.L., Kong X.Y., Liu P., Xin W.W., Fu L., Jiang L., Wen L.P., Qian Y.C. (2022). Bioinspired hier-archical porous membrane for efficient uranium extraction from seawater. Nat. Sustain..

[48] Yu R., Lu Y.R., Zhang X.S., Chen W., Chen X., Li L.B. (2022). Amidoxime-modified ultrathin polyethylene fibrous membrane for uranium extraction from seawater. Desalination.

[49] Yuan Y.H., Zhao S.L., Wen J., Wang D., Guo X.W., Xu L.L., Wang X.L., Wang N. (2019). Rational design of porous nanofiber adsorbent by blow-spinning with ultrahigh uranium recovery capacity from seawater. Adv. Funct. Mater..

[50] Xu X., Xu L., Ao J.X., Liang Y.L., Li C., Wang Y.J., Huang C., Ye F., Li Q.N., Guo X.J. (2020). Ultrahigh and economical uranium extraction from seawater via interconnected open-pore architecture poly (amidoxime) fiber. J. Mater. Chem. A.

[51] Pu Y.D., Qiang T.T., Ren L.F. (2022). Waste feather fiber based high extraction capacity bio-adsorbent for sustainable uranium extraction from seawater. Int. J. Biol. Macromol..

[52] Li B.Y., Liu J.Y., Chen S.S., Song Y., Liu Q., Yu J., Chen R.R., Zhu J.H., Li R.M., Wang J. (2024). A novel anti-biofouling collagen fiber grafted with hyperbranched polyethyleneimine/amidoxime for efficient uranium extraction from seawater. Desalination.

