# Supplementary material for: Anti-Biofouling Polyzwitterion–Poly(amidoxime) Composite Hydrogel for Highly Enhanced Uranium Extraction from Seawater
Source: Gels. 2024 Sep 22;10(9):603. doi: 10.3390/gels10090603 (PMC11431610; doi:10.3390/gels10090603)
Supplement: Supplementary file 1 [file gels-10-00603-s001.zip › gels-3197665-supplementary.pdf]

This PDF file includes:

Supplementary Materials and Methods

Figure S1 to S12

Table S1 to S5

References 1-19

Supporting Figures

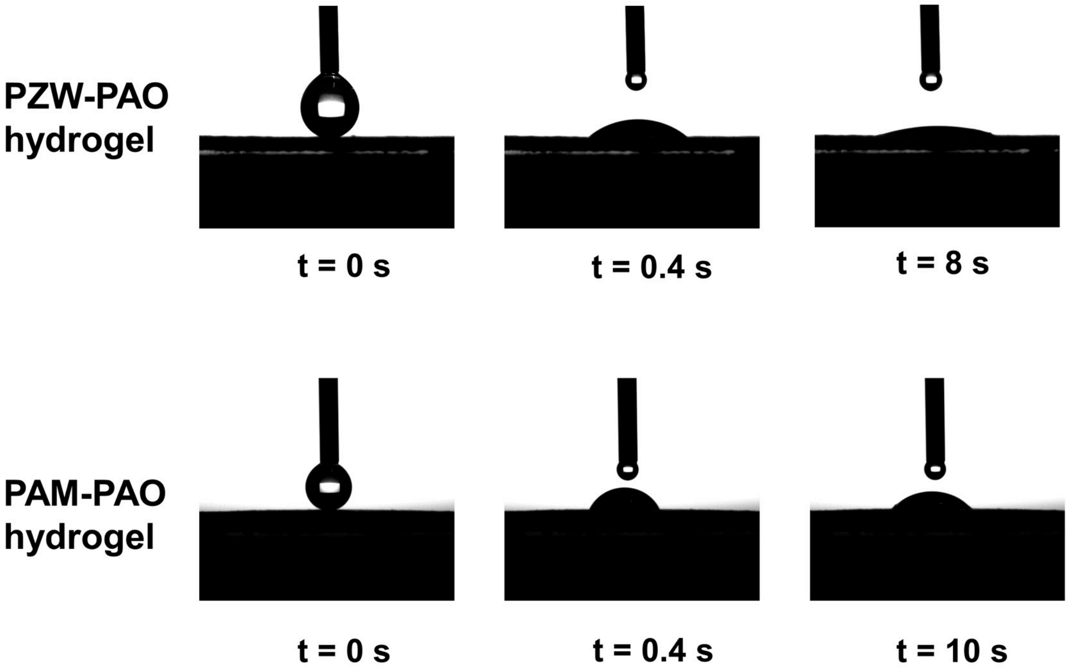

Figure S1. Comparison of the air/seawater contact angle change of the PZW-PAO hydrogel and the blank PAM-PAO hydrogel.

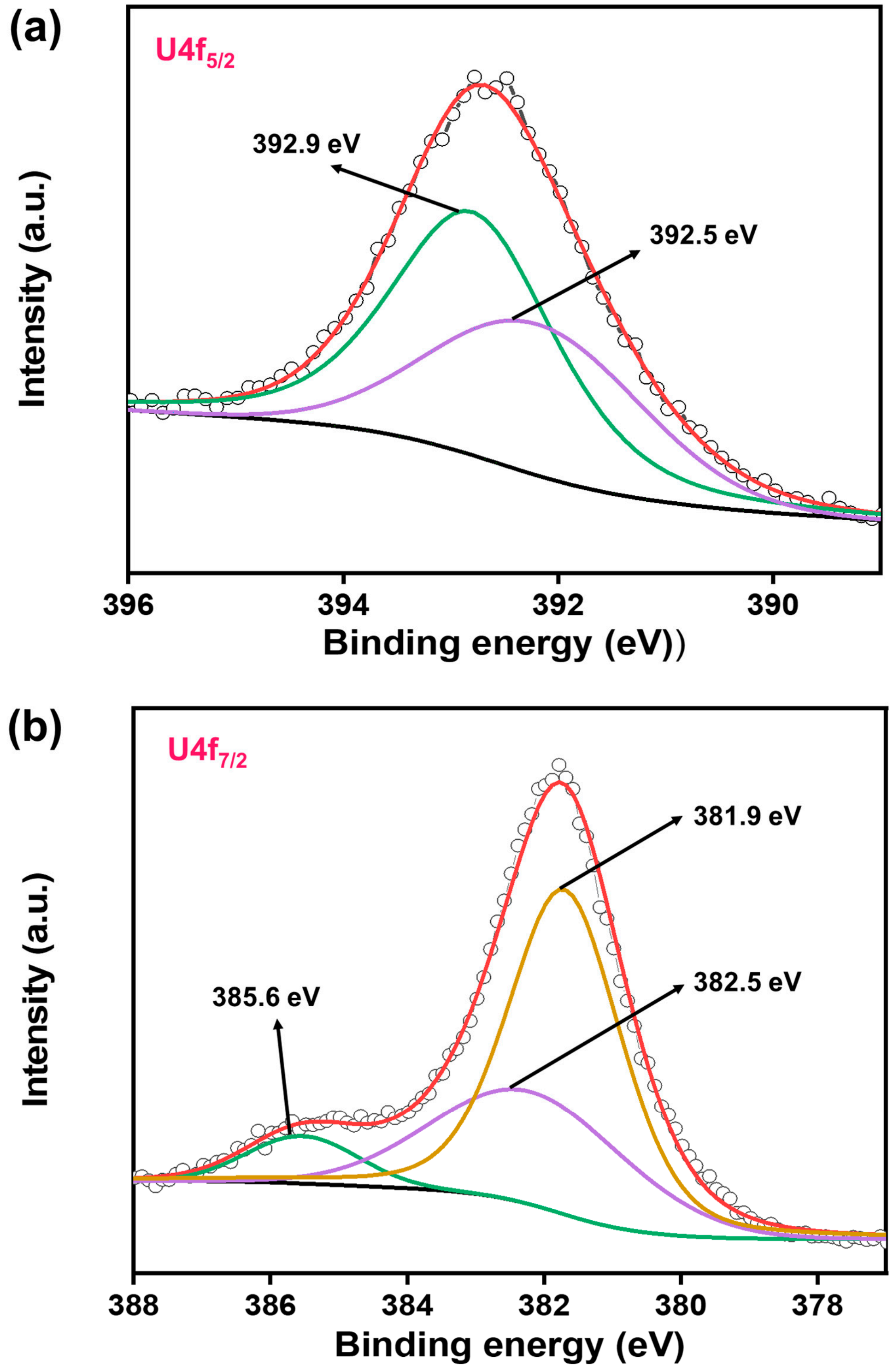

Figure S2. The split peak analysis of the U-uptake ZW-PAO hydrogel.

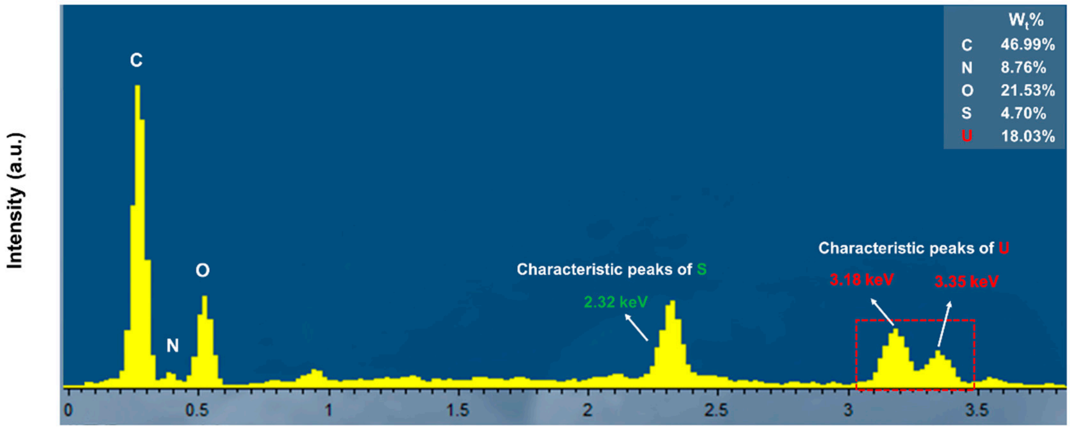

Figure S3. The EDS-mapping analysis of the U-uptake PZW-PAO hydrogel.

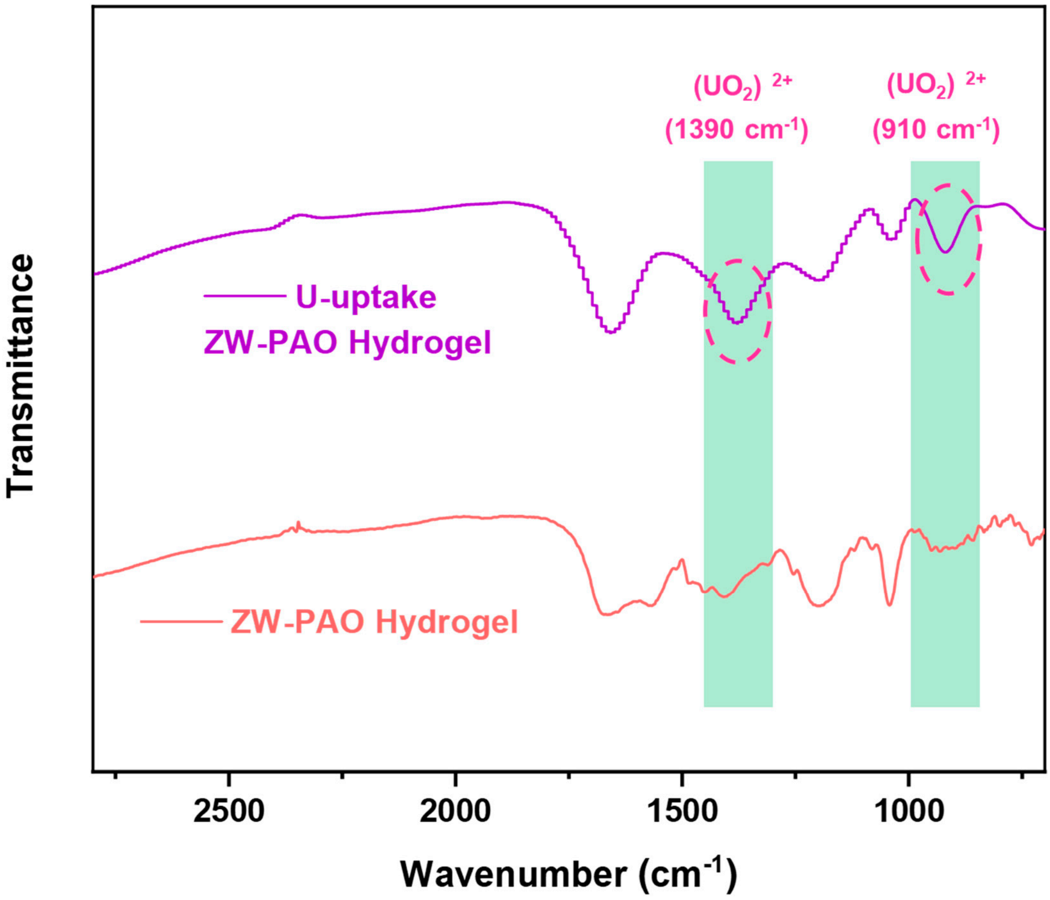

Figure S4. Comparison of FT-IR spectroscopy between the before and after U-uptake PZW-PAO hydrogel. [1,2].

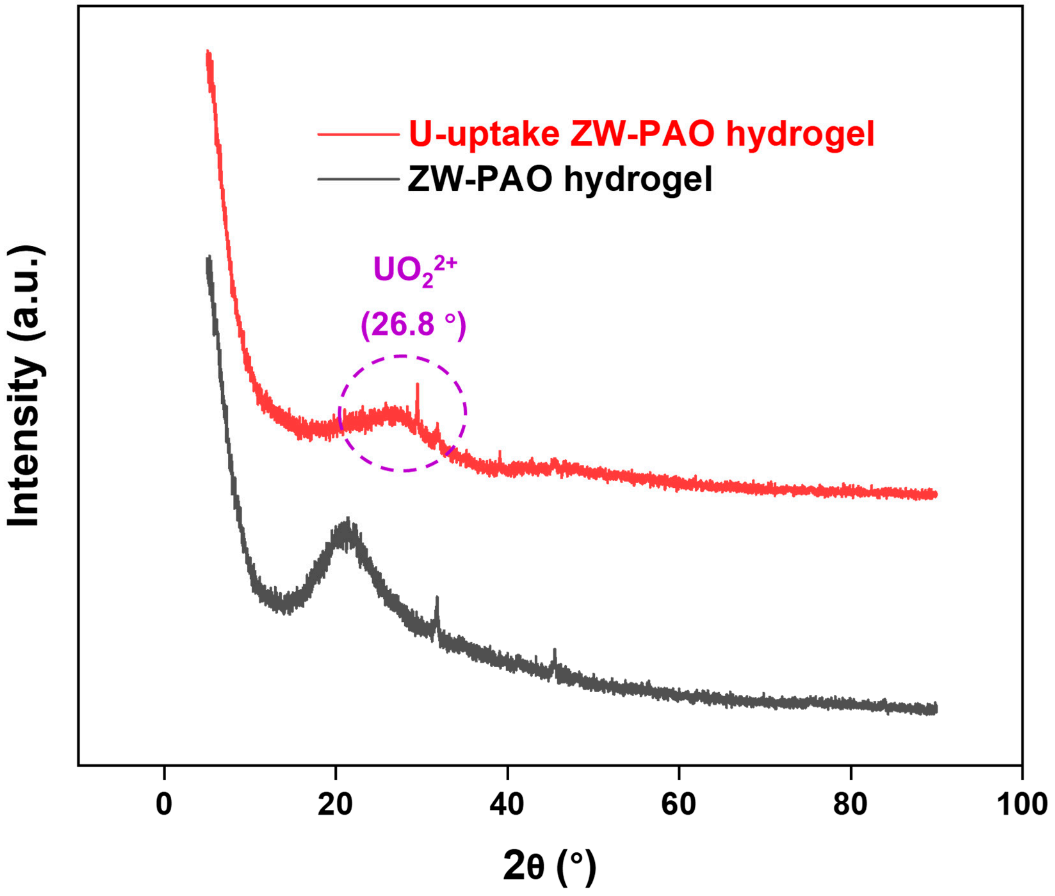

Figure S5. Comparison of the wide angle X-ray diffraction (WAXD) between the before and after U-uptake PZW-PAO hydrogel. [3].

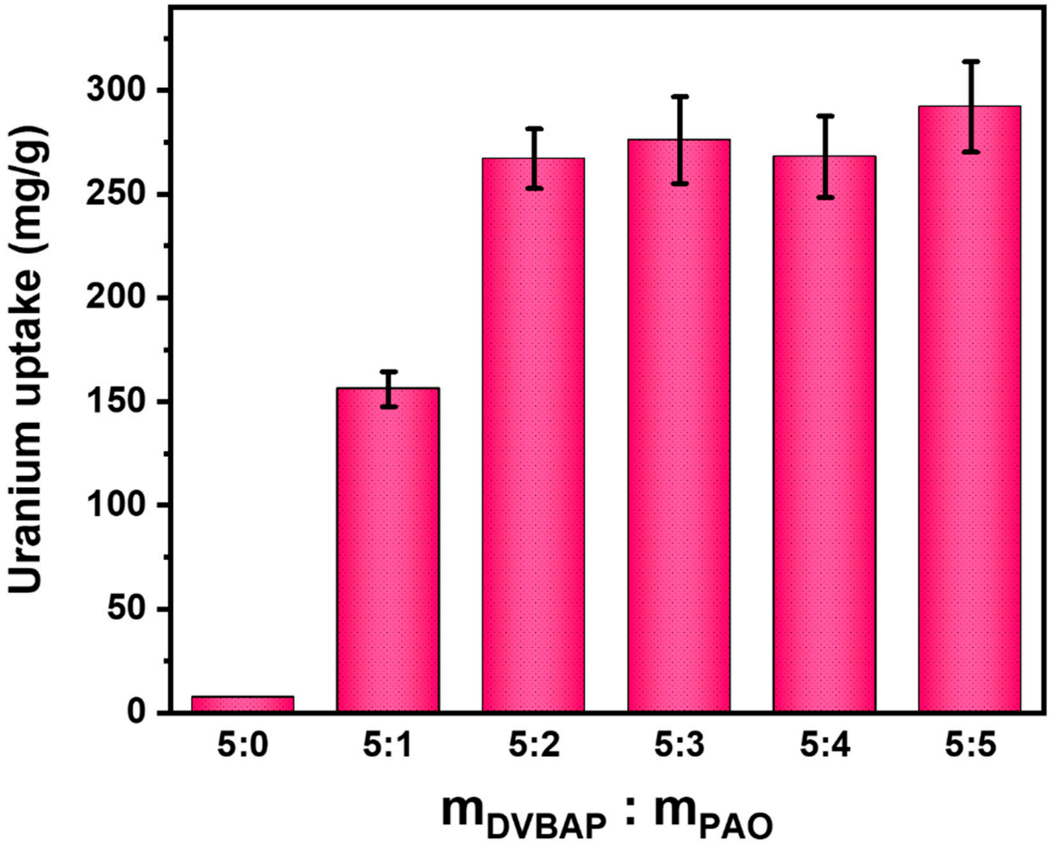

Figure S6. Uranium adsorption properties of hydrogels with different ratios (m_{DVBAP}: m_{PAO} = 5 : 0 to 5 : 5).

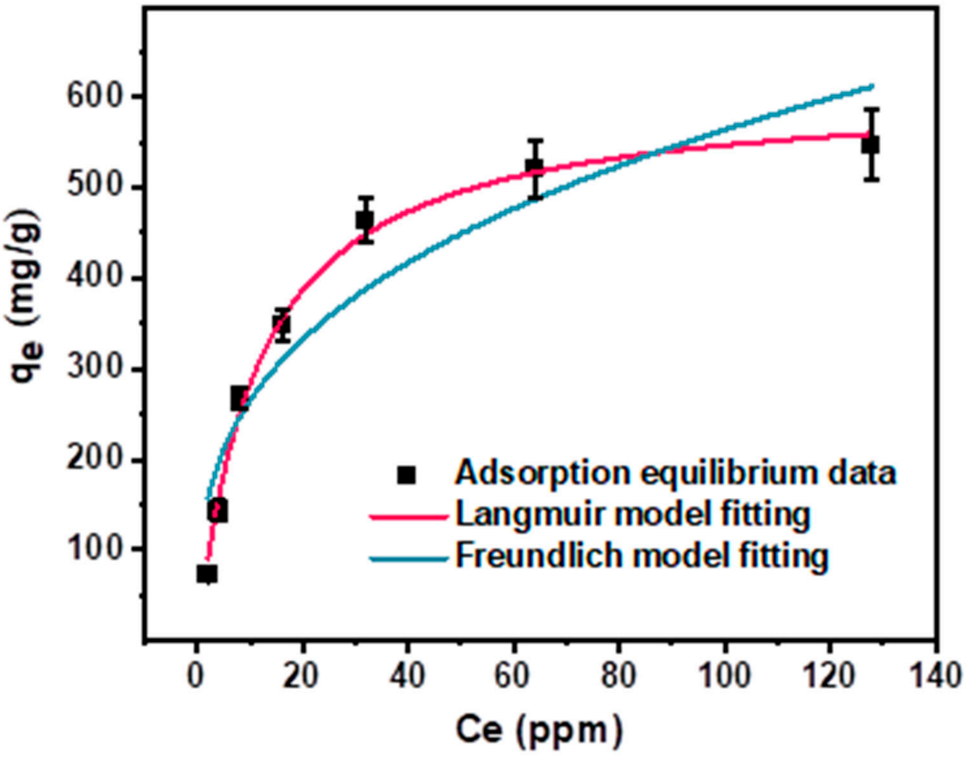

Figure S7. The adsorption thermodynamic curve of PAO-PZW hydrogel and Langmuir model and Freundlich model.

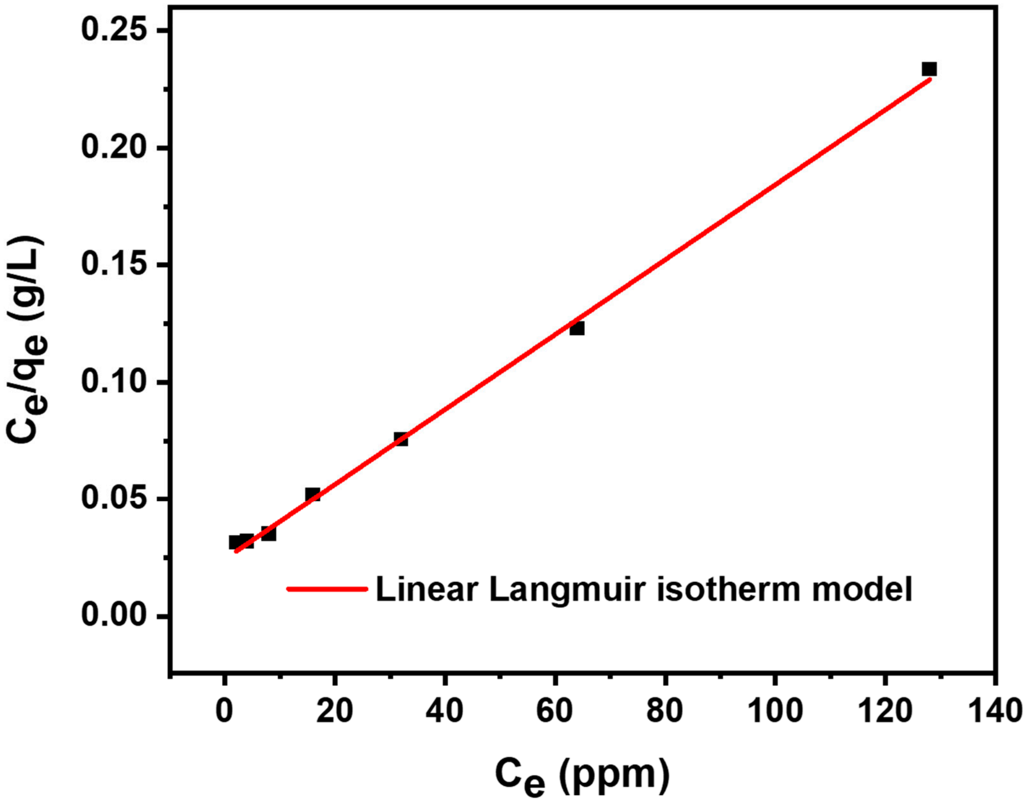

Figure S8. The linear Langmuir isotherm model for the U-adsorption.

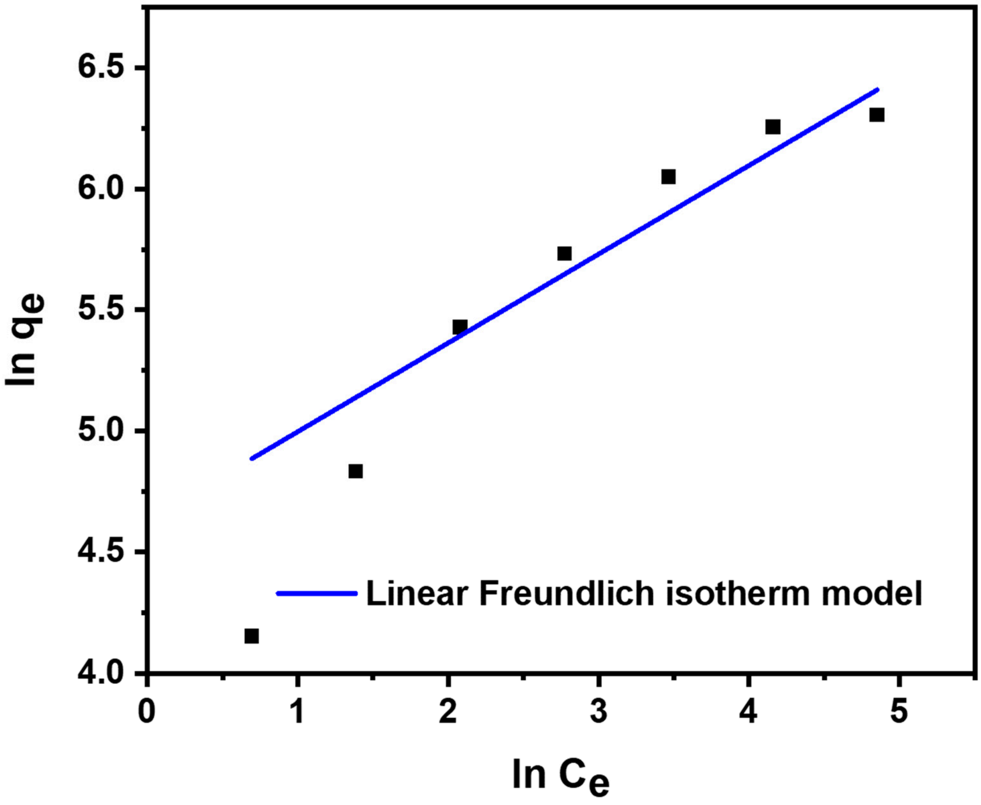

Figure S9. The linear Freundlich isotherm model for the U-adsorption.

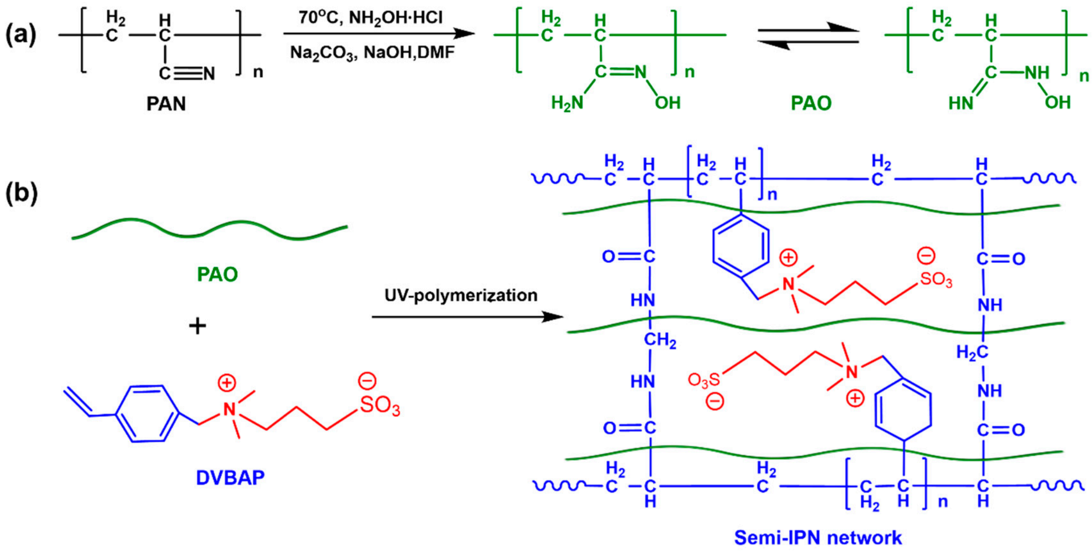

Figure S10. (a) Preparation of the PAO polymer. (b) The illustration on the fabrication and semi-interpenetrating structure of the ZW-PAO hydrogel.

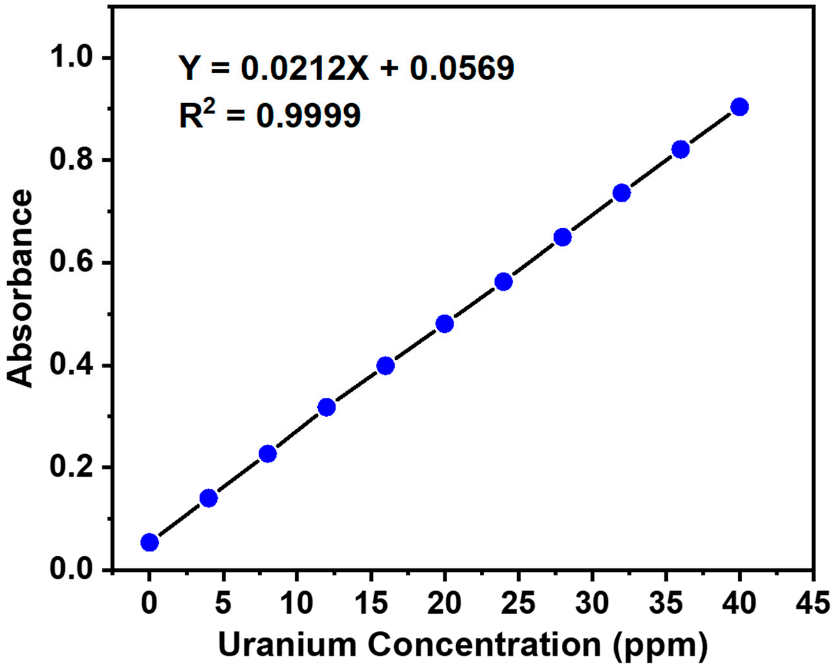

Figure S11. Standard curve for prediction of uranium concentration in pure water.

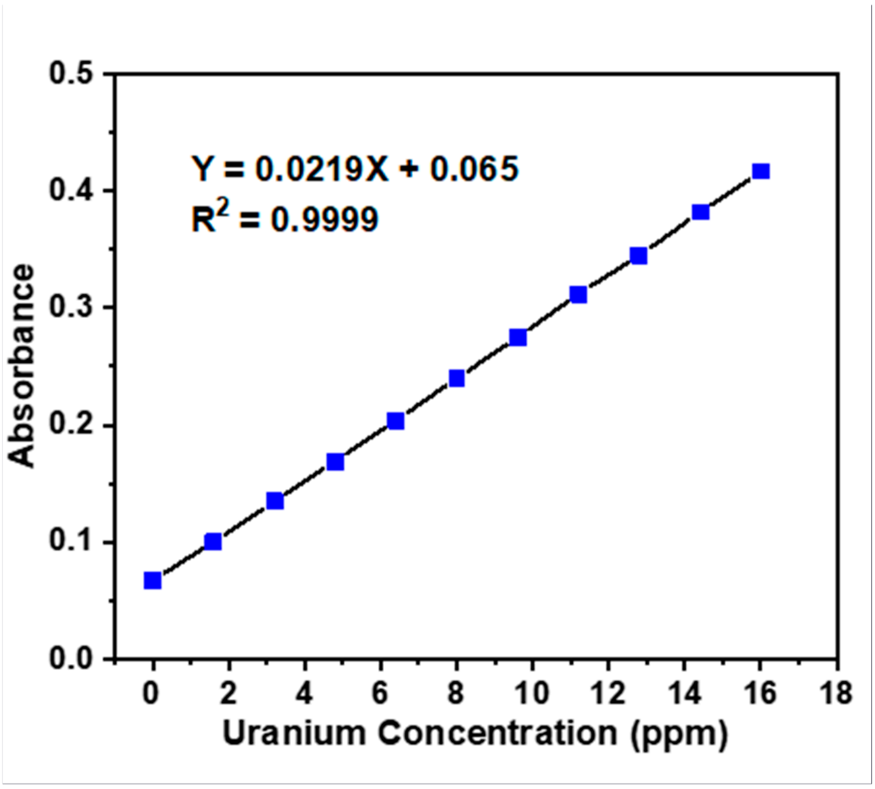

Figure S12. Standard curve for prediction of uranium concentration in seawater.

Table S1. Comparison of swelling ratio and water content of ZW-PAO hydrogel in pure water and seawater.

Sample	SR[a]	Water content (wt%) (wt%)	Dry gel content (wt%)
In water	238.54 ± 3.86	58.08 ± 0.73	41.92 ± 0.67
In seawater ssseawater	522.92 ± 8.19	80.87 ± 1.03	19.13 ± 0.21

a: SR = [$m_{hydrogel}$/$m_{dry\ gel}$] × 100%.

Table S2. PAO contents in different precursor solution of ZW-PAO hydrogels.

sample	PAO (mg)	DVBAPs (mg)	I-2959 (mg)	BIS (mg)
0	0	100	1	1
1	20	100	1	1
2	40	100	1	1
3	60	100	1	1
4	80	100	1	1
5	100	100	1	1

Table S3. The pseudo-second-order model for the uranium adsorption kinetics of ZW-PAO hydrogel in 2,4,8,16 ppm uranium-added seawater.

Pseudo-second-order model	R2	qe	k2 [g mg−1 min−1]
2ppm	0.9964	111	5.475×10-4
4 ppm	0.9975	198	3.940×10-4
8 ppm	0.9993	281	3.695×10-4
16 ppm	0.9996	391	2.269×10-4

Table S4. Concentration of U(VI) and co-existing metal ions in seawater and ×100 seawater.

Elements	concentration in seawater (ppb)	×100 concentration (ppb)	ion species
U	3.5	350	U(VI)
V	2.7	270	V(V)
Fe	1.4	140	Fe^{3+}
Ni	2.6	260	Ni^{2+}
Mn	0.4	40	Mn^{2+}
Co	0.4	40	Co^{2+}
Cu	1.9	190	Cu^{2+}
Na	8.25×106	8.25×106	Na^{+}
K	0.58×106	0.58×106	K^{+}
Ca	0.77×106	0.77×106	Ca^{2+}
Mg	1.14×106	1.14×106	Mg^{2+}

Table S5. Comparison of uranium extraction rate between ZW-PAO hydrogel and existing amidoxime group adsorbent.

Absorbents	U-uptake ($mg{\cdot}g^{-1}$)	Time (day)	U-uptake rate ($mg{\cdot}g^{-1}{\cdot}day^{-1}$)	Year	Ref.
**This work**	**9.38**	**25**	**0.375**		
Hydrogel	6.99	35	0.200	2020	[4]
Hydrogel	3.12	24	0.130	2023	[5]
Hydrogel	9.23	28	0.329	2020	[6]
Hydrogel	5.19	10	0.519	2022	[7]
Hydrogel	9.73	11	0.880	2024	[8]
Hydrogel	5.88	30	0.196	2021	[9]
Hydrogel	8.62	25	0.345	2023	[10]
Membranes	9.35	35	0.267	2020	[11]
Membranes	8.78	25	0.351	2021	[12]
Membranes	9.03	28	0.323	2022	[13]
Membranes	7.46	56	0.133	2022	[14]
Fibers	9.59	56	0.171	2020	[15]
Fibers	10.31	35	0.296	2019	[16]
Fibers	6.2	42	0.147	2020	[17]
Fibers	3.22	30	0.107	2022	[18]
Fibers	1.05	15	0.071	2024	[19]

Reference

1. Cao, H.; Bao, H.; Lin, X.; Lin, J.; Zhang, L.; Huang, Y.; Wang, J, Q. Differential interplay between Ce and U on local structures of U1-xCexO2 solid solutions probed by X-ray absorption spectroscopy. *J Nucl Mater*. **2019**, 515, 238-44.
2. Wang, W, G.; Zhang, S, L.; L, J. Infrared spectral characteristics of some common uranium minerals. *Chin J geol*. **1981,** 16(3), 235-46.
3. Qiu, L, F.; Ou, G, X.; Zhang, M.; Li, Q.; Wu, D.; Shang, C, J. Micro-area analysis of uranium minerals by Micro FT-IR spectrometry. *Acta Mineral Sin*. **2016**, 36(1), 43-7.
4. Bai, Z, Y.; Liu, Q.; Zhang, H, S.; Yu, J.; Chen, R, R.; Liu, J, Y.; Song, D, L.; Li, R, M.; Wang, J. Anti-biofouling and water—stable balanced charged metal organic framework-based polyelectrolyte hydrogels for extracting uranium from seawater. *ACS Appl. Mater. Interfaces*. **2020**, 12(15), 18012-22.
5. Zhang, J, Q.; Ma, J, L.; Jiao, G, J.; Liu, K, N.; Cui, R.; Zhai, S, R.; Sun, R, C. Methyl 4-hydroxybenzoate nanospheres anchored on poly (amidoxime)/polyvinyl alcohol hydrogel network with excellent antibacterial activity for efficient uranium extraction from seawater. *Desalination*. **2023**, 548, 116243.
6. Yan, B, J.; Ma, C, X.; Gao, J, X.; Yuan, Y, H.; Wang, N. Anion-crosslinked supramolecular hydrogel for ultrahigh and fast ura-nium recovery from seawater. *Adv Mater*. **2020**, 32(10), 1906615.
7. Zhang, C, R.; Cui, W, R.; Niu, C, P.; Yi, S, M.; Liang, R, P.; Qi, J, X.; Chen, X, J.; Jiang, W.; Zhang, L.; Qiu, J, D. rGO-based covalent organic framework hydrogel for synergistically enhance uranium capture capacity through photothermal desalination. *Chem. Eng. J*. **2022**, 428, 131178.
8. Liu, T.; Zhao, J, T.; Qiao, Q, T.; Zhang, R, Q.; Wei, T.; Liang, Y, X.; Yuan, Y, H.; Wang, N. Engineering shrinkage resistance of nano-structured hydrogels in seawater for fast uranium capture. *Chem. Eng. J*. **2024**, 153832.
9. Liu, R, R.; Wen, S, X.; Sun, Y.; Yan, B, J.; Wang, J, W.; Chen, L.; Peng, S, Y.; Ma, C.; Cao, X, Y.; Ma, C, X.; Duan, G, G. A nanoclay enhanced Amidoxime-Functionalized Double-Network hydrogel for fast and massive uranium recovery from seawater. *Chem. Eng. J*. **2021**, 422, 130060.
10. Song, Y, C.; Ma, X.; Tan, H, H.; Liu, Z.; Liu, C, T.; Shen, C, Y.; Yang, P, P.; Li, S, W. Hollow Zn/Co zeolitic imidazolate framework-implanted composite hydrogel for highly efficient uranium extraction from seawater. *Nano Res*. **2023**, 16(7), 10451-61.
11. Shi, S.; Qian, Y, X.; Mei, P, P.; Yuan, Y, H.; Jia, N.; Dong, M, Y.; Fan, J, C.; Guo, Z, H.; Wang, N. Robust flexible poly (amidoxime) porous network membranes for highly efficient uranium extraction from seawater. *Nano Energy*. **2020**, 71, 104629
12. Sun, Y.; Liu, R, R.; Wen, S, X.; Wang, J, W.; Chen, L.; Yan, B, J.; Peng, S, Y.; Ma, C.; Cao, X., Y; Ma, C, X.; Duan, G, G. Antibi-ofouling ultrathin poly(amidoxime) membrane for enhanced U(VI) recovery from wastewater and seawater. *ACS. Appl. Mater. Inter*. **2021**, 13(18), 21272-85.

13. Yang, L, S.; Xiao, H, Y.; Zhao, X, L.; Kong, X, Y.; Liu, P.; Xin, W, W.; Fu, L.; Jiang, L.; Wen, L, P.; Qian, Y,C. Bioinspired hier-archical porous membrane for efficient uranium extraction from seawater. *Nat. Sustain.* **2022**, 5(1), 71-80.
14. Yu, R.; Lu, Y, R.; Zhang, X, S.; Chen, W.; Chen, X.; Li, L, B. Amidoxime-modified ultrathin polyethylene fibrous membrane for uranium extraction from seawater. *Desalination*. **2022**, 539, 115965.
15. Li, Z.; Yu, Z, Q.; Wu, Y, D.; Wu, X, L.; Wan, Y.; Yuan, Y, H.; Wang, N. Self-sterilizing diblock polycation-enhanced polyami-doxime shape-stable blow-spun nanofibers for high-performance uranium capture from seawater. *Chem. Eng. J.* **2020**, 390, 124648.
16. Yuan, Y, H.; Zhao, S, L.; Wen, J.; Wang, D.; Guo, X, W.; Xu, L, L.; Wang, X, L.; Wang, N. Rational design of porous nanofiber adsorbent by blow-spinning with ultrahigh uranium recovery capacity from seawater. *Adv Funct Mater*. **2019**, 29(2), 1805380.
17. Xu, X.; Xu, L.; Ao, J, X.; Liang, Y, L.; Li, C.; Wang, Y, J.; Huang, C.; Ye, F.; Li, Q, N.; Guo, X, J.; Li, J, Y.; Wang, H, T.; Ma, S, Q.; Ma, H, J. Ultrahigh and economical uranium extraction from seawater via interconnected open-pore architecture poly (amidoxime) fiber. *J. Mater. Chem. A*. **2020**, 8(42), 22032-44.
18. Pu, Y, D.; Qiang, T, T.; Ren, L, F. Waste feather fiber based high extraction capacity bio-adsorbent for sustainable uranium extraction from seawater. *Int J. Biol. Macromol*. **2022**, 206, 699-707.
19. Li, B, Y.; Liu, J, Y.; Chen, S, S.; Song, Y.; Liu, Q.; Yu, J.; Chen, R, R.; Zhu, J, H.; Li, R, M.; Wang J. A novel anti-biofouling collagen fiber grafted with hyperbranched polyethyleneimine/amidoxime for efficient uranium extraction from seawater. *Desalination*. **2024**, 586, 117894.
